# OncodriveFML: a general framework to identify coding and non-coding regions with cancer driver mutations

**DOI:** 10.1186/s13059-016-0994-0

**Published:** 2016-06-16

**Authors:** Loris Mularoni, Radhakrishnan Sabarinathan, Jordi Deu-Pons, Abel Gonzalez-Perez, Núria López-Bigas

**Affiliations:** Research Program on Biomedical Informatics, IMIM Hospital del Mar Medical Research Institute and Universitat Pompeu Fabra, Doctor Aiguader 88, 08003 Barcelona, Catalonia Spain; Institució Catalana de Recerca i Estudis Avançats (ICREA), Passeig Lluís Companys 23, 08010 Barcelona, Spain

**Keywords:** Cancer drivers, Non-coding regions, Local functional mutations bias, Non-coding drivers

## Abstract

Distinguishing the driver mutations from somatic mutations in a tumor genome is one of the major challenges of cancer research. This challenge is more acute and far from solved for non-coding mutations. Here we present OncodriveFML, a method designed to analyze the pattern of somatic mutations across tumors in both coding and non-coding genomic regions to identify signals of positive selection, and therefore, their involvement in tumorigenesis. We describe the method and illustrate its usefulness to identify protein-coding genes, promoters, untranslated regions, intronic splice regions, and lncRNAs-containing driver mutations in several malignancies.

**Electronic supplementary material:**

The online version of this article (doi:10.1186/s13059-016-0994-0) contains supplementary material, which is available to authorized users.

## Background

Massive cancer genomic initiatives promoted by the advance of next generation sequencing technologies have uncovered the landscape of somatic mutations in cohorts of patients suffering from dozens of the most common tumor types [1, 2]. These initiatives, focused mostly on exome regions, demonstrated the pressing need to develop methods to identify protein-coding cancer drivers from the wealth of mutated genes in tumor genome [3]. Several methods that identify genes whose mutational patterns significantly deviate from the expected behavior have been developed and validated [3–8]. These abnormal mutational patterns are caused by positive selection acting on the mutations in driver genes during the course of tumorigenesis and tumor evolution [9]. A mutational frequency across a cohort of tumors that is significantly higher than the background mutation rate is the most intuitive and exploited signal of positive selection, implemented for example by computational approaches such as MuSiC [8] and MutSig [4]. Nevertheless, the correct estimation of the background mutation rate of protein-coding genes requires accounting for many known—and probably also yet unknown—covariates, some of which can currently only be approximated [10]. In 2012 we introduced a computational approach to detect a different signal of positive selection in driver genes: the bias towards the accumulation of high impacting mutations [5] across tumor samples. We proved that the top-ranking genes in their deviation of the average impact score of mutations observed from the expected average impact score of the same number of mutations in the given cohort—functional mutation (FM) bias—were *bona fide* cancer drivers.

Currently, the availability of hundreds of whole-genome sequences from tumors presents us with the unprecedented opportunity of identifying non-coding genomic regions involved in cancer development upon somatic mutations. Most of the approaches developed to scan protein-coding genes involved in tumorigenesis cannot be readily extended to the analysis of non-coding regions [3], and only few approaches designed to detect non-coding drivers have been recently proposed [11–14]. Moreover, catalogs of somatic mutations detected by targeted sequencing specific regions of genomes are accumulating as genome sequencing becomes routine in the clinical practice of oncology. The Memorial Sloan Kettering-Integrated Mutation Profiling of Actionable Cancer Targets (MSK-IMPACT) [15], for example, has carried out the targeted deep sequencing of all exons and selected introns of 341 key cancer genes of thousands of tumors every year (https://www.sloankettering.edu/blog/expanding-impact-precision-medicine-fuel-discoveries). Most current methods to detect drivers rely on whole-exome or whole-genome sequencing data to build their background models for analysis. Therefore, they are unable to mine this rich data accumulating from targeted sequencing to further pinpoint the regions within cancer genes that exhibit signals of positive selection in different tumor types.

Here we propose a novel approach, OncodriveFML, to estimate the accumulated functional impact (FI) bias of tumor somatic mutations in genomic regions of interest, both coding and non-coding, based on a local simulation of the mutational processes affecting it. OncodriveFML possesses two critical advantages over the current generation of methods aimed at identifying cancer drivers. First, it can directly compute the FM bias—and thus identify drivers—of any genomic element, provided only that a score to assess the FI of somatic mutations can be computed for that element. In the “Results” section, we illustrate this versatility by employing different FI scoring approaches to detect putative cancer drivers among: (1) coding genes; (2) intronic splicing regions; (3) promoter regions; (4) untranslated regions (UTRs) of messenger RNAs (mRNA); and (5) long non-coding RNAs (lncRNAs). This feature will prove crucial as more cohorts of cancer tumors are sequenced at the whole-genome range opening up the possibility to detect non-coding elements involved in tumorigenesis. Second, the FM bias of genomic elements can still be computed if only one or few of them have been sequenced across the cohort of patients, as is the case of targeted sequencing aimed at informing clinical decision-making. This raises the possibility of dramatically lowering the cost of sequencing cohorts of tumors to carry out analyses such as the identification of genes involved in specific cancer-related processes, such as drug resistance, metastasis, and tumor relapse. We provide the source code of a web server to detect putative driver genomic elements, both coding and non-coding on mutations data from cohorts of tumors using OncodriveFML (http://www.intogen.org/oncodrivefml).

## Results

### OncodriveFML computes a local FM bias

The rationale behind OncodriveFML is that the observation of somatic mutations on a genomic element (coding gene, promoter, UTR, lncRNA, etc) across tumors, whose average impact score is significantly greater than expected for said element constitutes a signal that these mutations have undergone positive selection during tumorigenesis. This, in turn is considered as a direct indication that this element drives tumorigenesis. We call this deviation of the observed average impact score of somatic mutation in a genomic feature from its expected value, the functional mutation bias, or FM bias. To measure the FM bias of a particular element, OncodriveFML is required to: (1) be able to compute a relevant score of the predicted impact of the mutations in the genomic element; and (2) simulate the mutational processes to compute the expected average impact score.

**Fig. 1:**
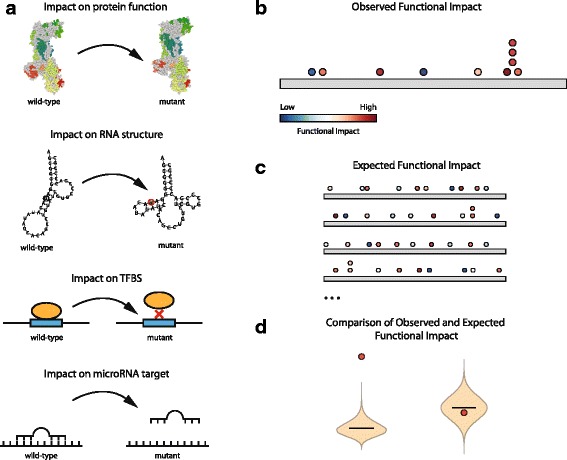
The OncodriveFML approach to detect signals of positive selection. **a** The functional impact (FI) of mutations may be computed in different manners for different types of genomic elements. **b** The FI of somatic mutations occurring in a genomics element across tumors are computed. **c** Mutation sets are randomly sampled from the element under analysis and the FI score of each simulated mutation is obtained. **d** The mean FI of the mutations observed in the element (*red dots*) is compared to the distribution of FI means of randomly generated mutations (*violin plots*) to obtain an empirical *p* value. On the *left* is shown an example of highly significant *p* value while the *violin plot* on the *right* illustrates a non-significant case

Any approach to compute the impact of mutations, however different depending on the type of genomic element under analysis, may serve the purpose of computing its observed FM bias (Fig. 1a). For instance, in mutations in protein-coding genes, one could measure the predicted impact on protein structure and function, while in RNA genes or UTRs, one could compute the impact of the mutations on RNA secondary structure, which is known to be key to their function. Also in UTRs, a useful measure of the FI of mutations could be their effect on the binding of microRNAs (miRNAs) to their target sites. In the case of promoters and enhancers, the effect of mutations on existing transcription factor binding sites or the creation of new ones may be assessed. Combined scores that take into account several features to measure the FI, such as CADD [16], may also be useful. The only requirements of the FI scoring approach is that it is relevant for the function of the genomic element under study and that it can be computed for all possible mutations in the element. Here we present implementations of OncodriveFML that use several FI scoring metrics.

OncodriveFML consists of three steps illustrated in Fig. 1b–d. In the first step, the average FI score of the set of somatic mutations observed in the element of interest across a tumor cohort is computed (Fig. 1b). In the second step, sets of mutations of the same size as the number of mutations observed in the element are randomly sampled from the universe of all the variants that it can possibly sustain (Fig. 1c). To accurately model mutational processes in the tumor type of interest, the sampling is done following the probability of mutation of different tri-nucleotides, which can either be computed from the mutations observed in each sample, in cohort under analysis as a whole, or pre-computed from previously analyzed tumor cohorts of the same or similar type. This random sampling is iterated a number of times (e.g. 10,000 times) to generate local expected average impact scores. Finally, OncodriveFML compares the average impact score observed for each element to its local expected average impact score resulting from the sampling and computes a local FM bias, in the form of an empirical *p* value which measures the deviation of the observed average score from the expected background (Fig. 1d). Elements with significant local FM bias after the correction for false discovery rate are deemed likely drivers.

### OncodriveFML detects driver coding genes

**Fig. 2:**
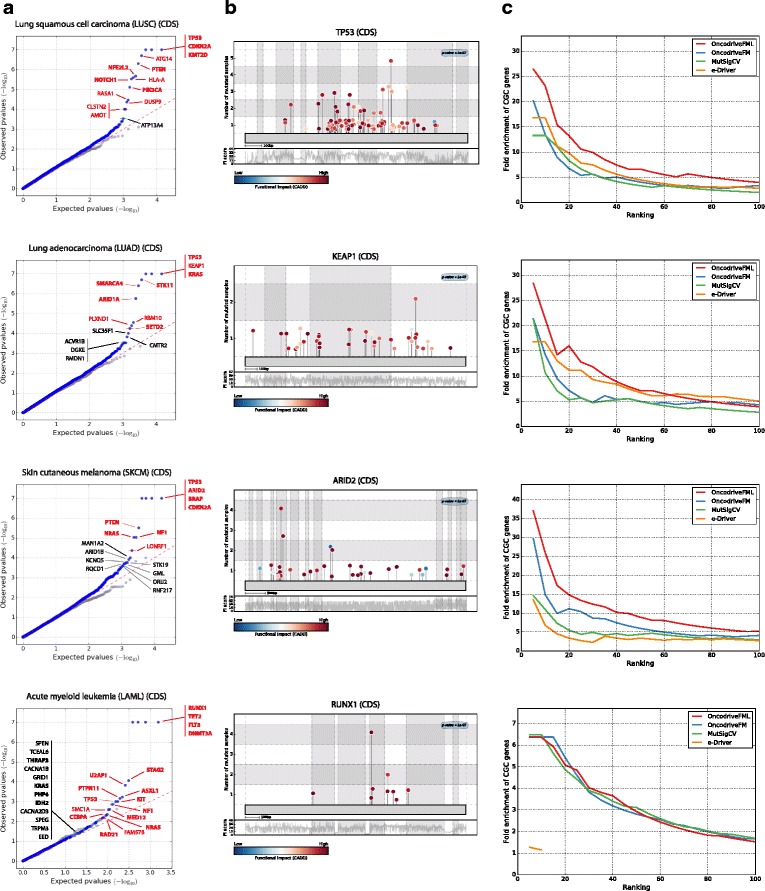
Results of the application of OncodriveFML to identify driver protein-coding genes across four cohorts of tumors. **a** *Quantile-quantile* (QQ) *plots* comparing the expected and observed distribution of FM bias *p* values of genes. *Gray dots* denote *p* values obtained on the randomized dataset that serves as negative control. Names in *red* indicate genes with FM bias q-value below 0.1, while names in *black* indicate genes with FM bias q-value below 0.25. Names in *bold* denote genes annotated in the Cancer Gene Census (CGC). **b** *Mutation needle-plots* showing the distribution of mutations along the sequences of the CDS of selected genes. The *color of the circles* follows the FI CADD score scale. The *y-axis* indicates the number of tumor samples in the cohorts where mutations at each position have been observed. The behavior of the CADD FI score across the entire CDS is shown below the *needle-plot*. **c** Fold increase in the proportion of CGC genes among sets with increasing number of top ranking genes detected by four methods: OncodriveFML, OncodriveFM, MutSigCV, and e-Driver. (See details in the text.) *QQ plots* and fold CGC proportion increase graphs for other 15 cohorts of tumors are available in Additional file 2, section A

To test its validity, we applied OncodriveFML to the detection of drivers among all human protein-coding genes using the set of somatic mutations detected by whole-exome sequencing across the tumors of 19 cohorts, (these and all datasets of somatic mutations employed here are described in Additional file 1). First, through quantile-quantile (QQ) plots comparing the expected and observed distribution of the FM bias *p* values (Fig. 2a and Additional file 2, section A), we demonstrate that the latter follows the expected homogeneous distribution of the null hypothesis, with the exception of the few cases that correspond to genes with significant FM bias. Among the top ranking genes identified by OncodriveFML in the four cohorts presented as examples in Fig. 2b and c, there are well-known cancer genes, such as TP53, KEAP1, ARID2, and RUNX1. Mutations observed in these genes exhibit a clear bias towards high FI (Fig. 2b; whole list in Additional file 3).

Second, as a proxy of the true positives rate of the method, we computed the fold enrichment in the proportion of known cancer genes (genes in the Cancer Gene Census (CGC) [17]) among its top ranking genes. We found that OncodriveFML performs better in this metric than the original version of OncodriveFM [5], MutSigCV [4], and e-Driver [18] (Fig. 2c and Additional file 2, section A) across the 19 cohorts of tumors analyzed. We also compared the results of OncodriveFML with a newer version of MutSigCV run by its authors [19], finding that there is an important overlap between the genes identified by both methods and that each method identifies additional true cancer genes missed by the other, stressing the complementarity of the two approaches (Additional file 2, section A). We next applied the OncodriveFML FM bias test to randomized datasets of mutations, built by reshuffling the mutations observed within each genome, following the rates of tri-nucleotides and the constraints of number of mutations per sample and per region. The number of genes detected as putative drivers within these random datasets of mutations would act as a proxy of the rate of false positive elements detected by the FM bias test. We carried out this analysis on the 19 whole-exome cohorts of tumors that constitute the WE-4482 dataset. OncodriveFML finds no significant gene in this dataset (gray dots in Fig. 2a), as expected for an accurate method with a low number of false positives. The whole list of driver candidates appears in Additional file 3. Taken together, the results presented in this section demonstrate that OncodriveFML identifies putative protein-coding driver genes with a sensitivity that outperforms five widely employed methods developed for this task, while maintaining a very low false positive rate.

### OncodriveFML detects driver non-coding elements

**Fig. 3:**
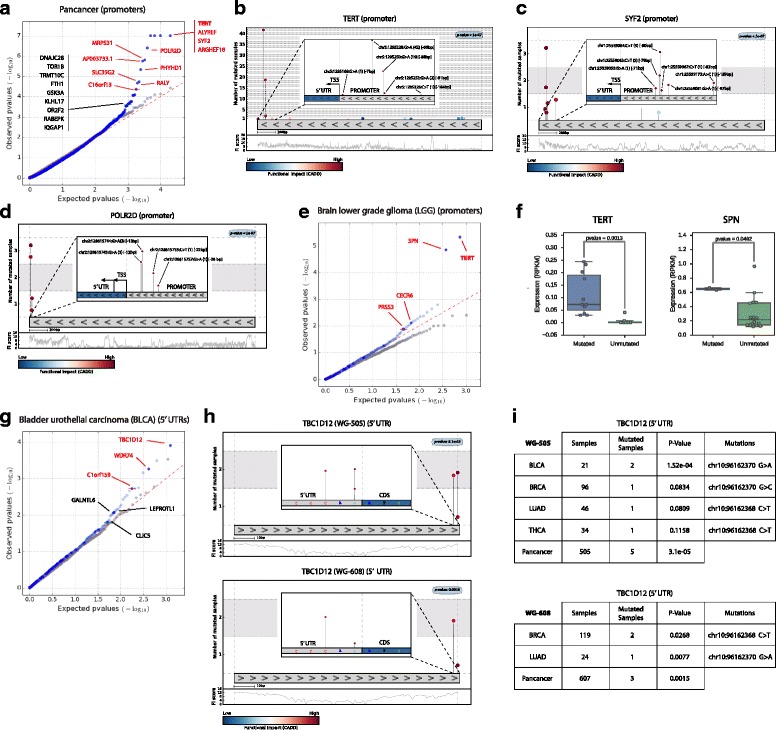
Results of the application of OncodriveFML to identify driver promoters and 5′ UTRs. The results of OncodriveFML are illustrated on mutations found across the pan-cancer cohort (**a**–**d**) and the cohorts of lower grade gliomas (**e**, **f**) and bladder urothelial carcinomas (**g**–**i**) of the WG-505 dataset. **a**, **e**, **g** *QQ plots* comparing the expected and observed distribution of FM bias *p* values of promoters and 5′ UTRs mutated in the respective cohorts. **b**–**d**, **h** *Mutation needle-plots* of selected promoters and 5′ UTRs, with a zoom at mutations located in the proximity of the transcription start site (TSS), or the 5 bps of the 5′ UTR closer to the CDS, respectively. **f** Comparison of the expression of two genes with significantly FM biased promoters in the cohort of lower grade gliomas in samples with mutations in the promoter and unmutated samples. In the *boxplots* the gene expressions of the mutated samples (*on the left*) is compared to those of unmutated samples (*on the right*). The expression values are reported in RPKM (Reads Per Kilobase of transcript per Million mapped reads) on the *y-axis* and the number of samples (mutated and normal) in each set are indicated with *dots* on the *boxplots*. The significance of the differential expression between mutated and non-mutated samples is reported at the top of each plot (Wilcoxon rank-sum test). I. Significance of the 5′ UTR of the TBC1D12 gene across several cohorts of both the WG-505 and WG-608 datasets

**Fig. 4:**
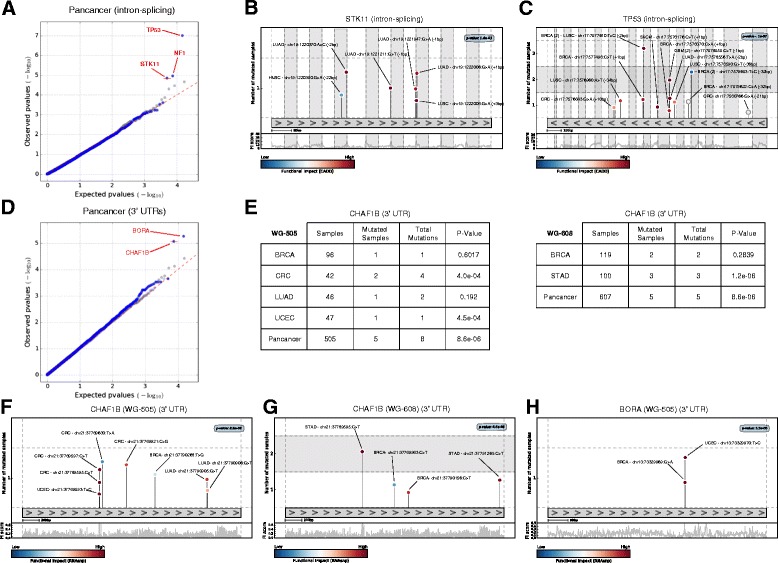
Results of the application of OncodriveFML to identify driver splice intronic regions and 3′ UTRs. The results of OncodriveFML are illustrated on mutations found across the pan-cancer cohort of the WG-505 dataset. **a**, **d** *QQ plots* comparing the expected and observed distribution of FM bias *p* values of splice intronic regions and 3′ UTRs mutated in the pan-cancer cohort. **b**, **c**, **f**–**h** *Mutation needle-plots* of selected splice intronic regions and 3′ UTRs. **e** Significance of the 3′ UTR of the CHAF1B gene across several cohorts of both the WG-505 and WG-608 datasets

One of the most interesting features of OncodriveFML is its applicability to the detection of driver non-coding genomic element. Therefore, we next tested its performance in the identification of putative driver promoter, 5′ UTR, splice intronic, and 3′ UTR regions of coding genes containing mutations across 22 tumor cohorts with whole-genome data sequenced by TCGA [12] or other projects [20] (datasets WG-505 and WG-608, respectively, in Additional file 1), as well as two pan-cancer cohorts resulting from pooling the mutations detected in all cohorts of each dataset (Figs. 3 and 4). Our primary aim was to test OncodriveFML at the identification of putative driver non-coding elements and compare its performance with other two recently published methods to the same effect [13, 14]. Due to the complete absence of a curated gold standard of non-coding driver elements, we limited the comparison to the assessment of the rate of false positives detected by each method through the analysis of the QQ plots of their observed and expected distributions of *p* values and the analysis of randomized datasets. We used CADD to score the FI of mutations occurring in all aforementioned non-coding elements, with the exception of 3′ UTRs, where we used the score provided by RNAsnp to that effect (see below). As with coding genes, the observed and expected distributions of the FM bias *p* values of the 22—and pan-cancer—cohorts correlate very well, and when applied to randomized mutation dataset it shows a good control of false positives (Fig. 3a). In this regard, OncodriveFML compares favorably with two recently published methods in the identification of putative non-coding elements across the cohorts of both the WG-505 and the WG-608 datasets (Additional file 2, section B). In the following sections, we describe in detail the most interesting candidate drivers of each type of non-coding elements identified as significantly FM biased.

### OncodriveFML uncovers putative driver promoters and 5′ UTRs

In the pan-cancer cohort, comprising 505 tumors the promoter regions with the most significant FM bias comprise a shortlist of interesting candidate drivers, such as those of TERT (details in Fig. 3b), SYF2, ARGHEF18, and POLR2D. TERT encodes the reverse transcriptase subunit of telomerase required to maintain telomere length during tumor growth. Activating mutations in TERT promoter have been found as drivers in multiple types of cancer [12, 21, 22]. Other promising novel candidates include the promoters of SYF2 (details in Fig. 3c), an mRNA splicing factor thought to interact with a cell cycle regulator [23], ARHGEF18, a Rho-specific guanine nucleotide exchanging factor involved in RhoA activation and cell motility [24], and POLR2D (Fig. 3d), a subunit of the RNA polymerase, which contains mutations very close to the transcription start site (TSS) in melanoma samples [25, 26].

In a cohort of 18 lower grade gliomas, in addition to the TERT promoter, OncodriveFML identifies the promoter of SPN with a highly significant FM bias (Fig. 3e). The expression of both genes is significantly higher in samples bearing mutations in their promoters than in non-mutated samples (Fig. 3f), which provides foundation to the idea that these mutations are positively selected during tumorigenesis. SPN is normally produced solely by white blood cells where it regulates functions such as cell-cell adhesion, intracellular signaling, apoptosis, migration, and proliferation. Its ectopic expression in solid tumors has been reported and proposed as a target for immunotherapy [27]. In summary, in addition to producing a very low rate of false positive results in the detection of putative driver promoter elements, OncodriveFML is able to identify already known and putative interesting promoters with driver mutations, even at very low mutational frequency. The whole list of putative driver promoters (and other non-coding elements) appears in Additional file 3.

Among top-ranking FM biased 5′ UTR regions, we found that of TBC1D12 (Fig. 3g). This gene encodes a GTPase-activating protein for Rab family proteins. 5′ UTR mutations are recurrently found near the start codon (Fig. 3h) and are detected in samples of different tumor types. We detected it also as FM biased in cohorts of the WG-608 dataset (Fig. 3i). The proximity of the mutations to the start codon suggests that they could have an effect on translational control. These mutations were recently reported as significantly redundant by [28], with 15 % of bladder tumor samples bearing mutations using whole-exome data.

### OncodriveFML uncovers genes with driver mutations in splice intronic regions

We next analyzed with OncodriverFML the intronic regions of coding genes, specifically, the 50 bps from exon-intron boundary (intron-50 bps) using CADD as a functional scoring framework to identify genes with driver intron-splicing mutations. In the pan-cancer cohort, of the WG-505 dataset, OncodriveFML identifies a shortlist of well-known tumor suppressor genes—TP53, STK11, and NF1—as highly FM biased in their intron-splicing mutations (Fig. 4a-b). TP53 contains 16 mutations within the first 50 bps of its introns, seven of which appear in breast cancer samples, while the others are distributed across the cohorts of other tumor types (including GBM, CRC, LUSC, SKCM, LUAD) (Fig. 4b). Interestingly, eight of these mutations are within the first 2 bps of the intron-exon boundary. STK11 is a serine/threonine-protein kinase known to act as a tumor suppressor in the control of the activity of AMP-activated protein kinase (AMPK) family members, thereby playing a role in various processes such as cell metabolism, cell polarity, apoptosis, and DNA damage response, often bearing inactivating mutations in in lung adenocarcinomas [29, 30]. Most of the mutations (4 out of 6) observed in the pan-cancer cohort of the WG-505 dataset falling within the first 50 bps of its introns indeed correspond to lung adenocarcinoma samples and all are in close proximity to the intron-exon boundary (Fig. 4c).

### OncodriveFML identifies putative driver 3′ UTRs

Next, we employed OncodriveFML to identify driver genes upon mutations in their 3′ UTR regions. In this case, we used the impact of mutations on RNA secondary structure computed by RNAsnp as FI score to compute the FM bias of mutations [31]. In the pan-cancer cohort of the WG-505 dataset, OncodriveFML identified BORA and CHAF1B as putative driver genes from the mutations in their 3′ UTR regions (Fig. 4d–h). Mutations contributing to the computed FM bias for CHAF1B in the WG-505 dataset appear in BRCA, CRC, LUAD, and UCEC. On the other hand, in the pan-cancer cohort of the WG-608 dataset, where it also appears as significantly FM biased, mutations appear in BRCA and STAD samples (Fig. 4e). CHAF1B is a chromatin assembly factor implicated in DNA replication and DNA repair [32]. BORA is an Aurora kinase activator, involved in the maturation of the centrosome, the assembly of the spindle and asymmetric protein localization during mitosis [33].

### OncodriveFML identifies putative lncRNAs

We next employed OncodriveFML to explore the potential of a group of lncRNAs collected from the literature the biological functions of which have been established [34–36] (Additional file 4). The mutated lncRNAs among these (across cohorts in the WG-505 and WG-608 datasets) were thus analyzed by OncodriveFML and those significantly FM biased in at least one cohort appear in Additional file 4. As in the case of 3′ UTRs, we computed the FM bias using an FI metric that estimates the impact of mutation on the RNA secondary structure [31]. We found that MALAT1, a lncRNA gene previously shown to be involved in tumorigenesis of lung adenocarcinomas [37], exhibits a slightly significant FM bias in cohorts of both the WG-505 (*p* value 0.0138 in KIRC) and the WG-608 (*p* value 0.0104 in pan-cancer) datasets. In addition, we detected a higher than expected accumulation of high functional impacting somatic mutations in MIAT, a non-protein-coding transcript associated with myocardial infarction in the WG-505 dataset (*p* value 0.0281 in CRC and *p* value 0.0163 in pan-cancer).

### OncodriveFML detects positive selection from the sequence of a gene panel

**Fig. 5:**
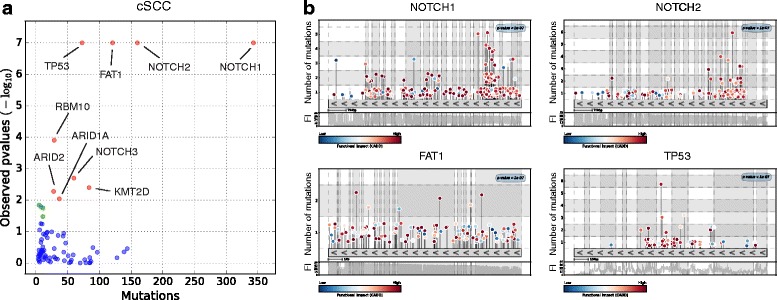
Results of the application of OncodriveFML to the somatic mutations identified in a panel of genes in 234 biopsies of normal skin. **a** *p* value vs. number of mutations of the 74 genes sequenced in the panel. Genes identified as significant with a q-value <0.1 (*red dots*) are indicated by their name while genes identified as significant with a q-value <0.25 are marked as *green dots*. **b** *Mutation needle-plots* of the most significant genes

Finally, we analyzed the list of somatic mutations detected in a panel of genes sequenced at high coverage across 234 biopsies of sun-exposed epidermis [38] to illustrate the use of OncodriveFML on the task of detecting genes under positive selection in the case when most mutations in the sample are unavailable to the method. OncodriveFML detects nine genes with a FM bias q-value <0.1 (Fig. 5a and b), which include the five genes identified by the authors using a modified *dn*/*ds* approach (NOTCH1, NOTCH2, FAT1, TP53, RBM10) and four other *bona fide* drivers of tumorigenesis (NOTCH3, ARID2, KMT2D, ARID1A). Six out of these nine genes are detected as drivers of cutaneous squamous cell carcinoma (cSCC), the tumor type that develops more frequently upon the malignization of sun-exposed epidermis. The results reveal the potential of OncodriveFML in identifying genes under positive selection among those sequenced as part of a panel.

We have made OncodriveFML available to cancer genomics researchers both to download and install the code and to run through a web application (http://www.intogen.org/oncodrivefml).

## Discussion

OncodriveFML introduces the construction of a local mutational background to compute the FM bias of genomic elements, a fundamental innovation which differentiates it from other methods designed to pick up signals of positive selection in genes across tumor samples. The local background model ensures that factors influencing the mutation rate in the large-scale—such as chromatin compaction or replication timing—do not result in over or underestimation of the FM bias of any genomic element. Other factors that affect the background mutation rate locally may still disturb the calculation of the FM bias of genomic elements and, as they are discovered, they should be incorporated into this local modeling, as is the case of transcription factor binding sites [39]. The combination of this local-background-building approach and the compliance with the mutational signature in the construction of the background model for the FM bias result in very fine-tuned statistical test that produces a very low rate of false positives, effectively outperforming the current generation of methods to detect driver genomic elements, both coding and non-coding, as we have demonstrated in this article. Furthermore, in principle OncodriveFML could also be used to detect negative selection in the pattern of somatic or germline variants observed across cohorts of samples taken from either tumors or normal tissues.

Although the accuracy of OncodriveFML relies on the predicted FI of mutations, these predictions need not be accurate enough to correctly identify individual driver variants, because the method computes the average FI of mutations and compares it to the distribution of all possible mutations in the region. Since FI scores are more developed for coding mutations (as is the case with CADD, which includes more information to score coding mutations), OncodriveFML may currently lose non-coding driver regions where the FI of mutations is poorly assessed. Nevertheless, the development of new methods aimed at scoring non-coding mutations as more whole genomes of tumors are sequenced will improve the quality of OncodriveFML in non-coding regions. Furthermore, in all the results shown in this paper in non-coding genomic elements, the FM bias has been calculated exclusively based on nucleotide substitutions. Finding a way of accurately scoring the FI of indels in non-coding regions will probably contribute to uncover new putative driver non-coding elements.

As the application of next generation sequencing technologies moves into the clinic, sequencing panels of genes—or any genomic regions—rather than the whole exomes or whole genomes of cohorts of tumors will become the most frequent choice [40]. Sequencing gene panels can also have important applications in research to detect subsets of known cancer genes that act as drivers or play other roles, such as drug resistance in specific cohorts of cancer patients. Here we have demonstrated that due to the computation of the local FM bias of genes, OncodriveFML is capable of detecting genes undergoing positive selection even if the catalog of all exomic mutations is not available for analysis. This capability will prove critical to analyze the results of sequencing panels.

## Conclusions

Here we describe OncodriveFML, a novel method able to detect putative cancer driver genomic regions through the computation of a local FM bias. As we have shown, OncodriveFML is able to operate on both coding and non-coding genomic regions, or any combination thereof to reveal their involvement in tumorigenesis. Furthermore, due to its unique local background model of the FI of mutations it is able to detect signals of positive selection in tumorigenesis when only the sequence of a small portion of the genome is available, opening the opportunity to exploit data obtained from the interrogation of gene panels.

## Methods

### Tumor mutations datasets

We analyzed a dataset of somatic mutations detected by whole-exome sequencing (dataset WE-4482), two datasets of somatic mutations detected by whole-genome sequencing (datasets WG-608 and WG-505), and a dataset of somatic mutations detected in a panel of genes sequenced at high coverage (dataset GP-234). The first dataset (WE-4482) contains the somatic mutations of 19 cancer types, the results of four of which (lung squamous cell carcinoma, lung adenocarcinoma, skin cutaneous melanoma, and acute myeloid leukemia) are shown in Fig. 2. The sources of the dataset are listed in Additional file 1. The second dataset (dataset WG-608) contains the somatic mutations of seven cancer types (breast invasive carcinoma, chronic lymphocytic leukemia, liver cancer, lung adenocarcinoma, lymphoma B-cell, medulloblastoma, and pilocytic astrocytoma) extracted from the work by Alexandrov et al. [20] plus a supplementary stomach adenocarcinoma dataset [41]. The third (dataset WG-505) contains the somatic mutations of 14 cancer types determined by TCGA [12]. The fourth dataset (dataset GP-234) contains the somatic mutations detected in a panel of genes sequenced with high coverage across 234 biopsies of sun-exposed epidermis [38]. The datasets were filtered to discard all possible false positives of the somatic mutations calling and we restricted the analyses to single nucleotide substitutions (SNV) excluding from the study both insertions and deletions. The number of samples and the number of mutations for each dataset are listed in Additional file 1.

### Definition of the coordinates of genomic elements

The genomic coordinates of both coding genes and lncRNAs were obtained from the ENCODE website (http://www.gencodegenes.org/), using Gencode release 19. Specifically, the coordinates of genes were retrieved from ftp://ftp.sanger.ac.uk/pub/gencode/Gencode_human/release_19/gencode.v19.annotation.gtf.gz. We only consider CDS of genes where both the “gene_type” and the “transcript_type” metadata were annotated as “protein-coding.” We defined intronic splice sites as 50 bp regions at the edges of protein coding introns. We removed from the datasets of UTRs all overlapping coding regions and short intronic splice sites of 10 bp. In addition, we removed from 5′ UTRs any overlap with 3′ UTRs. Promoter regions were defined as sequences 2500 bp upstream the TSS of protein-coding genes. We also removed from the dataset of promoters any region previously annotated as protein-coding (CDS), as untranslated region (3′ UTR and 5′ UTR), or as short intronic splice site. Overlapping elements of the same type (CDS of the same gene, UTRs of the same gene, intronic splice sites of the same gene, or promoters of the same gene) were merged together. Finally, we manually discarded genomic elements that show any evidence of erroneous annotation.

The genomic coordinates of lncRNAs were obtained from ftp://ftp.sanger.ac.uk/pub/gencode/Gencode_human/release_19/gencode.v19.long_noncoding_RNAs.gtf.gz. We only considered the exons of lncRNAs where both the “gene_type” and the “transcript_type” metadata were annotated as “lincRNA.” We removed from the lncRNAs any region previously annotated as protein-coding (CDS), as untranslated region (3′ UTR and 5′ UTR), or as short intronic splice site. and we merged together overlapping exons of the same lncRNA.

### Functional impact scoring metrics used in the analysis

The FI scores of all possible nucleotide changes in CDS and promoters of genes were retrieved from the CADD framework [16] version 1.0 (http://krishna.gs.washington.edu/download/CADD/v1.0/whole_genome_SNVs.tsv.gz). This framework provides a score of deleteriousness for every possible substitution of each nucleotide of the human genome and can consequently be applied to every coding or non-coding element of the genome. In the case of 3′ UTRs and lncRNAs, the FI for every possible substitution in each position was calculated using RNAsnp [31] to predict the impact of mutation on RNA secondary structure. The RNAsnp was executed with the “Mode 1” option (with other parameters set to default) to compute the local structural effect of mutations. For our analysis, we retrieved the resultant Euclidean distance score, which represents the difference between the base pair probabilities of wild-type and mutant ensemble structures as the basis to compute the FM bias.

### OncodriveFML methodological details

OncodriveFML has the ability to use different scoring frameworks, such as CADD [16] or RNAsnp [31]. Since the method is independent of the scoring system used, new scores can easily be incorporated to it as soon as they become available.

For every coding or non-coding element OncodriveFML proceeds with the following steps:

1. It first retrieves the FI score of all the mutations that can occur in an element (for instance, a gene or a UTR). The FI scores will vary depending on the scoring framework used (CADD, RNAsnp, etc.).
2. It then calculates the mean of the FI scores of all the observed mutations in the element across tumors.
3. It randomly takes from the pool of scores of all possible nucleotide changes in the element under analysis N samples of the same size as the number of observed mutations. The higher the N, the more resolution the derived empirical *p* values will have. For every random-generated mutation, its FI score is retrieved from the framework in use. The random sampling procedure can vary depending on the probability of each mutation to be sampled:
  - It can be assumed that all the random generated mutations have equal probability to occur.
  - It can be assumed that the probability of each mutation to occur is based on the mutational signatures (i.e. tri-nucleotide composition) observed in the dataset. For the analysis presented in this study we computed matrices of tri-nucleotide probabilities of the 96 possible changes (e.g. probability of ATC to mutate to ACC, probability of ATC to mutate to AGC, etc.). The matrices are computed by counting all the observed mutations in all the tumors. The random sampling process will thus take into account the different probabilities that each mutation has to occur. For instance, if a dataset show a higher frequency of the mutation ATT to ACT, the same mutation will be more likely to be sampled.
4. It computes the mean of each of the N random-generated group of mutations. N random sampling will generate a vector of N mean of FI scores.
5. It computes an empirical *p* value by comparing the mean of the observed FI scores with the distribution of means of the randomized FI scores. In brief, it counts how many times the mean of the observed FI scores is bigger than the means of the randomized FI scores, and then normalizes this value by the number of randomizations performed (N). For instance, if 10,000 randomizations have been computed (N is 10,000) and only two of these values are bigger than the mean of the observed FI scores, then the empirical *p* value would be 2/10,000, or 2*10–4. If the resulting *p* value is equal to 0, the number of randomizations N can be increased in order to gain a better *p* value resolution. The obtained *p* value indicates how likely the mean of the observed FI scores is expected by chance.
6. The resulting *p* values are then adjusted with a multiple testing correction using the Benjamini–Hochberg procedure. In the present study, we adjusted the *p* values of regions mutated in at least two samples in individual tumor types and five samples in the pan-cancer dataset.

### Comparison of OncodriveFML to other methods in the identification of putative driver coding genes

The somatic mutations used in the comparison were detected by whole-exome sequencing across the tumors of 19 cohorts (4482 samples in total). The sources of the data along with the number of samples and the number of mutations of each cancer type are listed in Additional file 1. The list of known cancer genes (CGC) [17] were retrieved from the Catalog Of Somatic Mutations In Cancer (COSMIC): sftp://sftpcancer.sanger.ac.uk/files/grch38/cosmic/v72/cancer_gene_census.csv. MutSigCV (version 1.4) was run using default parameters and using the full coverage file and the genes covariates provided by the authors. OncodriveFM was run using default parameters but setting the gene threshold (i.e. minimum number of mutations per gene to compute the FM bias) to 1. e-Driver was run with the mode option set to 1 (DOM). To compare OncodriveFML with the most recent version of MutSigCV, not publicly available at the time of writing, we obtained the list of significant genes identified by MutSigCV as run directly by the authors from the web http://tumorportal.org/. Since *p* values are not provided, in this case it was not possible to compute the proportion of known cancer genes among the top ranking genes as in Fig. 2c, and instead we compared the set of overlapping genes between both methods (Additional file 2, section A). Further details of these comparisons appear in the text in Additional file 2.

### Generation of datasets of random mutations

For each dataset of somatic mutations analyzed, we created a corresponding random dataset containing the same number of samples and the same number of somatic mutations (per sample) as the original dataset. The simulated mutations were randomly repositioned within windows of 50 Kb maintaining the original mutational signature, i.e. the same tri-nucleotide composition. The mutations whose reported reference allele was different from the corresponding position in the reference genome (hg19) were not included in the simulation.

### Needle plots

Figures 2, 3, 4, 5 show the linear distribution of mutations along the sequence of a genomic element (gene, UTR, promoter, etc.). The positions of the mutations correspond to their relative location inside the element. If an element is fragmented in different segments, these are concatenated and the fragments are represented with vertical dashed lines. The y-axis, and thus the height of the dots, indicates how many mutations have been observed in a given position (i.e. number of samples that share the same mutation) and the color of the dots represents the functional impact (FI) score of each mutation in the element. The color scale is normalized by the range of scores presents in the element after considering all the possible mutations. The “>” and “<” signs inside the element denote its strand. The highest possible FI score of each position of the element is represented at the bottom of the needle plot. The *p* value of the element is reported at the top-right of the plot.

### Expression analysis

Precomputed expression data were retrieved from Fredriksson et al. [12]. A detailed explanation of how the expression level were determined is provided in the original paper. In short, the authors obtained the RNA-sequencing (RNA-seq) and copy-number data (Affymetrix SNP6) from the cgHub repository (https://cghub.ucsc.edu). Gene expression level were subsequently determined by processing of raw sequencing data in BAM format considering the coding and lncRNA subsets of the GENCODE (v17) annotation and using HTSeq-count [42] as described in Akrami et al. [43]. Copy-number amplitudes were determined from segmented data (Affymetric SNP6 platform) available from TCGA by considering the minimum amplitude of all overlapping copy-number segments for each gene.

The significance of the differential expression between mutated and non-mutated samples was assessed using a Wilcoxon rank-sum test. For a given gene and cancer type, the test was performed only if at least 25 % of tumors had a detectable expression (i.e. non-zero value). In addition, the copy-number alteration data were taken into account and copy number-altered samples were excluded from the analysis (i.e. absolute amplitude expressed in log2 scale >0.2). After applying the filtering, we required at least ten remaining samples per gene and cancer type in order to compare the expression of the two groups.

## Abbreviations

CADD, combined annotation dependent depletion; CGC, Cancer Gene Census; COSMIC, Catalog Of Somatic Mutations In Cancer; cSCC, cutaneous squamous cell carcinoma; FI, functional impact; FM bias, functional mutations bias; GP-234, dataset of a gene panel sequence of 234 tumor samples; TCGA, The Cancer Genome Atlas; WE-4482, dataset of whole-exome sequence of 4482 tumor samples; WG-505, dataset of whole-genome sequence of 505 tumor samples; WG-608, dataset of whole-genome sequence of 608 tumor samples

## Additional files

Additional file 1: An Excel file with a description of all datasets of mutations used to test the performance of OncodriveFML in the detection of putative driver genomic elements. Each tab in the file contains the description of a separate dataset, with the first tab serving as a README. (ODS 14 kb) Additional file 2: A.pdf file containing a thorough description of methods and results of the performance of OncodriveFML in comparison to other common methods aim at the detection of driver genomic elements. Section A describes the comparison with tools aimed at detection driver coding genes; section B describes the same comparison in the case of non-coding drivers detection tools. (PDF 4957 kb) Additional file 3: An Excel file containing all significantly FM biased genomic elements, both coding and non-coding detected across all mutational datasets analyzed. The file is organized in tabs each corresponding to the results in one dataset. (ODS 57 kb) Additional file 4: An Excel file containing information on lncRNAs probed for FM bias. The first sheet contains a README file with details on the contents of the other two sheets. The second sheet contains the list of all lncRNAs probed, a summary of their biological function, and the original source from which we extracted them. The third sheet contains the FM bias *p* value computed for each significantly FM biased lncRNA in each cohort. (ODS 13 kb)
