# Supplementary material for: OncodriveFML: a general framework to identify coding and non-coding regions with cancer driver mutations
Source: Genome Biol. 2016 Jun 16;17:128. doi: 10.1186/s13059-016-0994-0 (PMC4910259; doi:10.1186/s13059-016-0994-0)
Supplement: Additional file 2: — A.pdf file containing a thorough description of methods and results of the performance of OncodriveFML in comparison to other common methods aim at the detection of driver genomic elements. Section A describes the comparison with tools aimed at detection driver coding genes; section B describes the same comparison in the case of non-coding drivers detection tools. (PDF 4957 kb) [file 13059_2016_994_MOESM2_ESM.pdf]

Benchmarking OncodriveFML in comparison with other tools to detect putative driver elements

Two main problems hinder the comparison of the performance of OncodriveFML with other methods aimed at detecting cancer driver genomic features. First, the lack of a gold standard of experimentally validated driver and non-driver features determines the use of catalogs of driver elements curated from the literature by experts. This problem becomes specially acute in the case of non-coding drivers (see below). Second, some methods to detect drivers (as is often the case in bioinformatics) are difficult to run either because the most up-to-date code is unavailable or due to lack of documentation and maintenance. In this document, we describe the paths we have taken to overcome these two difficulties and the results of the comparison of the performance of OncodriveFML and other tools designed to detect driver genomic elements.

A) Benchmark of the detection of driver coding genes

We benchmarked the performance of OncodriveFML in comparison to 3 other methods aimed at detecting driver coding genes, namely OncodriveFM (Gonzalez-Perez & Lopez-Bigas, 2012), e-Driver (Porta-Pardo & Godzik, 2014), and MutSigCV (Lawrence et al., 2013). The comparison to methods able to analyze only a small subset of mutated genes, as is the case of OncodriveCLUST (Tamborero, Gonzalez-Perez, & Lopez-Bigas, 2013), were deemed not meaningful. The somatic mutations used in the comparison were detected by whole-exome sequencing across the tumors of 19 cohorts (4482 samples in total). The sources of the data along with the number of samples and the number of mutations of each cancer type are listed in Additional data file 1. MutSigCV (version 1.4) was run using default parameters and using the full coverage file and the genes covariates provided by the authors. OncodriveFM was run using default parameters but setting the gene-threshold (i.e. minimum number of mutations per gene to compute the FM bias) to 1. e-Driver was run with the mode option set to 1 (DOM). In these comparisons we focused on two main properties of the outcome of the methods. First, we analyzed the relationship between the observed and expected distribution of p-values of genes produced by each method using quantile-quantile plots (qq-plots). Briefly, we plotted the -log10 of the observed p-values on the y axis and the -log10 of the expected p-values on the x-axis. The rationale behind the qq-plot is that if the null hypothesis tested by the method is correct and tests on genes are independent, the vast majority of p-values observed should coincide with those expected, that is predicted by the null hypothesis. In the graphs in Figure 2A of the main manuscript and Figures 1 to 4 below, the blue dots representing the p-values of genes should follow the red dashed line, which represents the homogeneous distribution of p-values. Only a small set of cases –genes whose behavior greatly deviates from the null hypothesis and which therefore behave significantly in the test– should appear away from the red line.

To guarantee that all methods had been run on the same datasets to ensure their comparability, we ran the programs ourselves on the 19 TCGA mutations cohorts of the dataset WE-4482, representing the same number of tumor types. The results of MutSigCV on all the cohorts are presented in Figure 1 of this document, those of e-Driver and OncodriveFM are presented in Figures 2 and 3 respectively, and the results of OncodriveFML are presented in Figure 4. (The results on four of the cohorts appear also in Figure 2A of the main manuscript.) In the case of e-Driver, which computes a p-value for each domain of the protein, we selected only the domain with the lowest p-value for each protein to build the qq-plots.

Notably OncodriveFML and MutSigCV are the only methods in the comparison that exhibit a calibrated distribution of p-values that is neither inflated nor deflated, suggesting that their rate of false positives/false negatives is kept lower than in other methods. This constitutes an evidence that the accuracy of OncodriveFML is fully satisfactory.

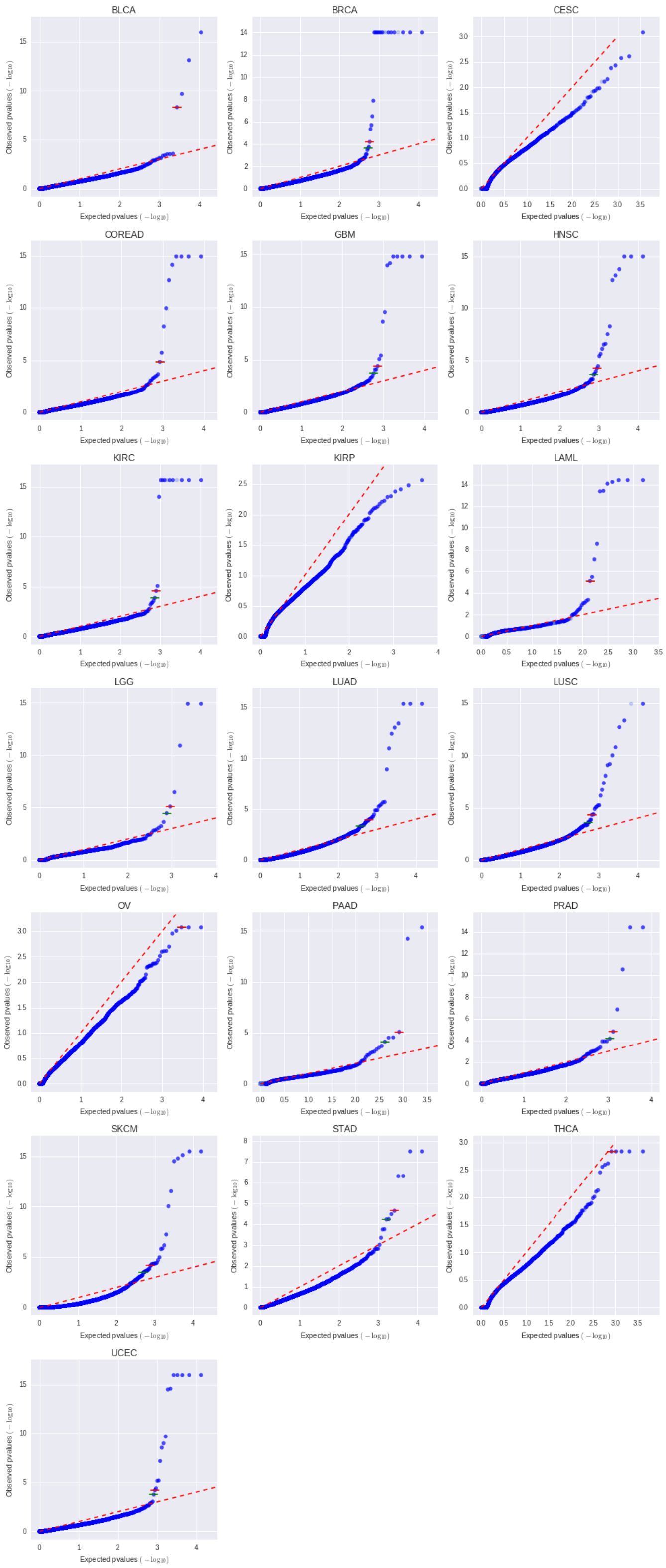

Figure 1. Quantile-quantile (qq) plots comparing the distribution of observed and expected p-values of tests carried out by MutSigCV on mutations in coding genes in 19 cohorts of tumors. False discovery rate thresholds (0.1 and 0.25) are indicated as short red and green segments in each graph.

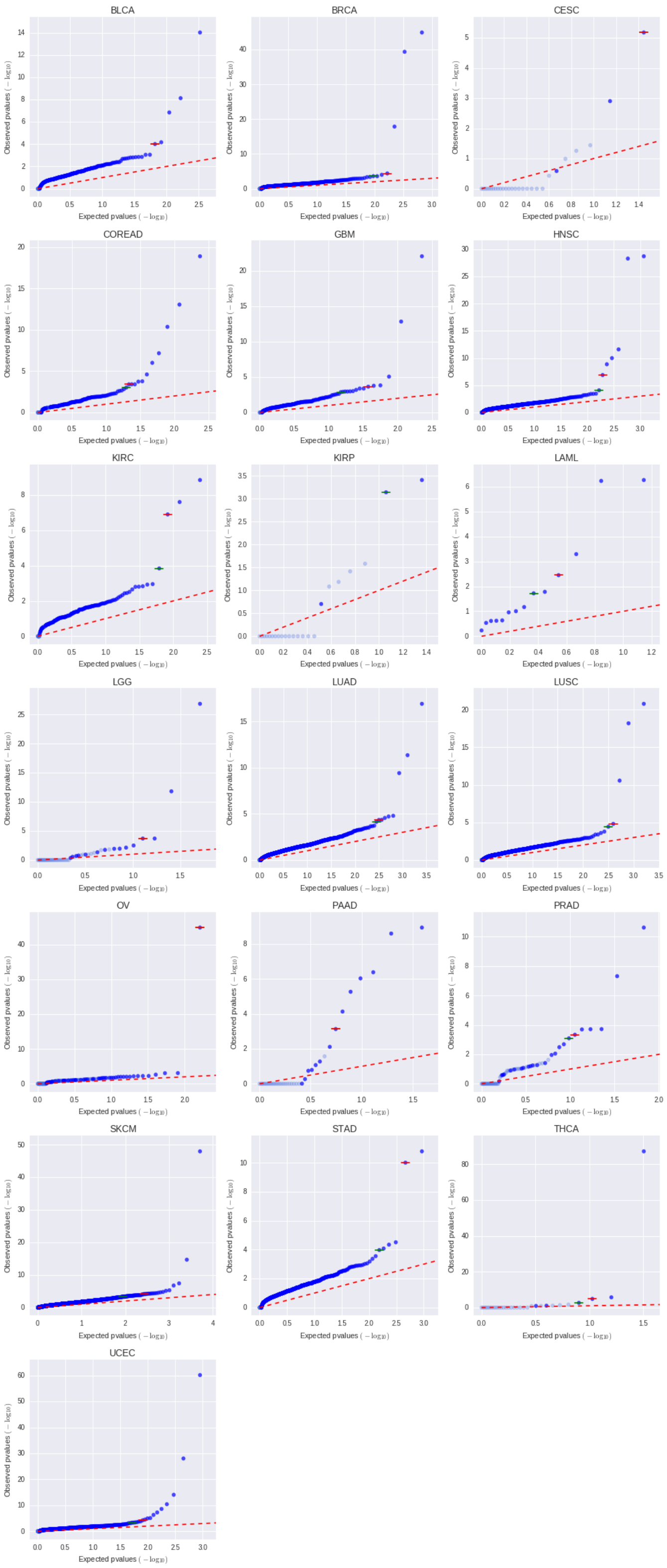

Figure 2. Quantile-quantile (qq) plots comparing the distribution of observed and expected p-values of tests carried out by e-Driver on mutations in coding genes in 19 cohorts of tumors. False discovery rate thresholds (0.1 and 0.25) are indicated as short red and green segments in each graph.

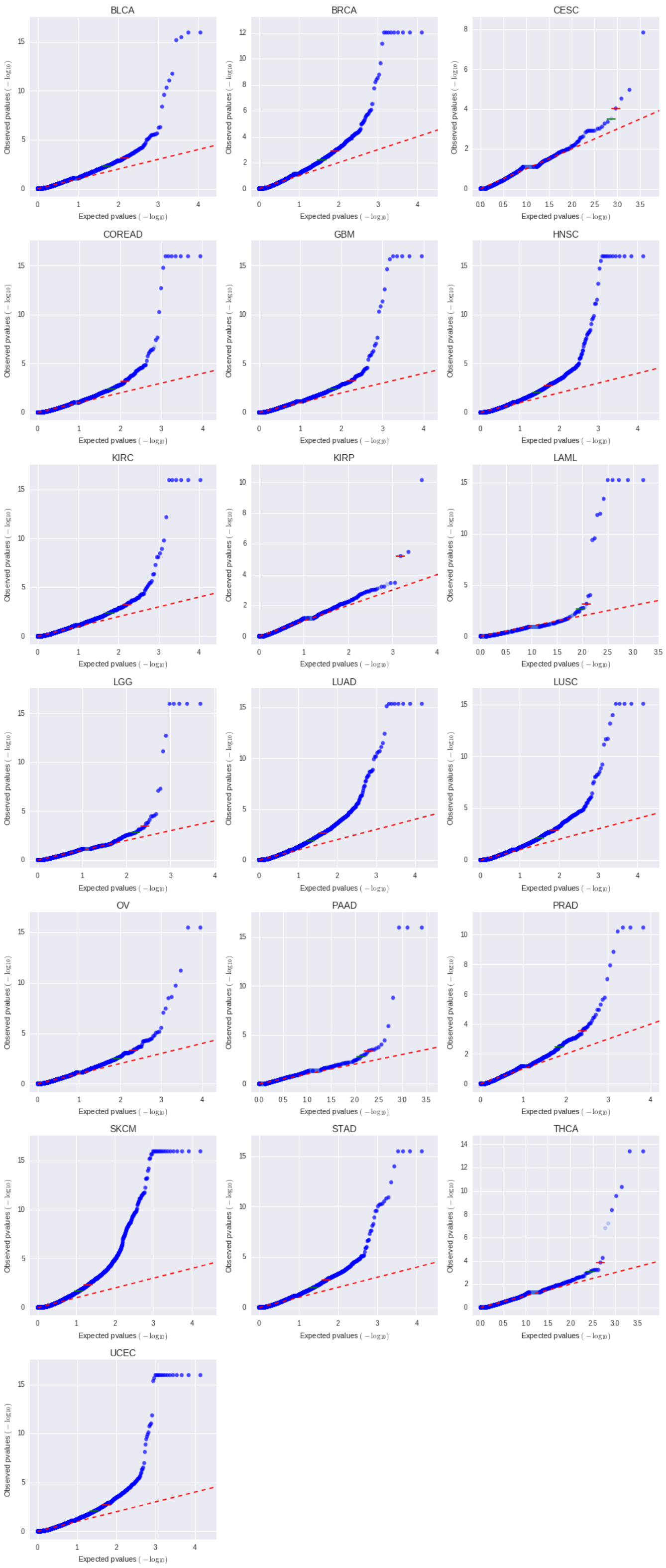

Figure 3. Quantile-quantile (qq) plots comparing the distribution of observed and expected p-values of tests carried out by OncodriveFM on mutations in coding genes in 19 cohorts of tumors. False discovery rate thresholds (0.1 and 0.25) are indicated as short red and green segments in each graph.

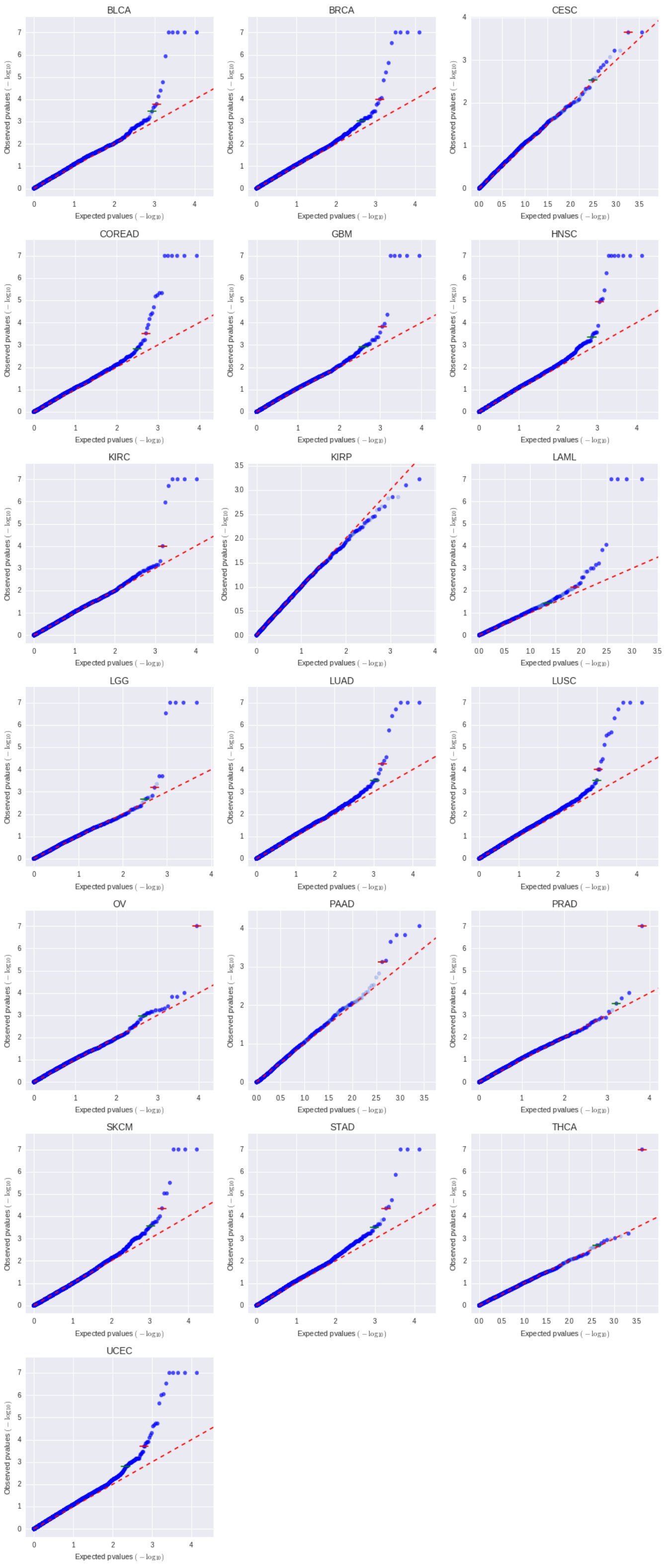

Figure 4. Quantile-quantile (qq) plots comparing the distribution of observed and expected p-values of tests carried out by OncodriveFML on mutations in coding genes in 19 cohorts of tumors. False discovery rate thresholds (0.1 and 0.25) are indicated as short red and green segments in each graph.

In a second comparison of the performance of the methods of the detection of driver coding genes, we computed the enrichment for CGC genes across the top ranking genes identified by each method. To do this, we computed the quotient between the ratio of CGC genes within these growing sets of top ranking genes and the fraction of CGC genes within all genes probed by the method. The denominator of this quotient corrects for the fact that some methods probe only a fraction of coding genes which may be already enriched for CGC genes. We carried out this analysis on the same nineteen cohorts as before. The results for four of these cohorts are presented in Figure 2C of the main manuscript; results of the remaining 15 cohorts appear in Figure 5A of this document.

In all datasets analyzed OncodriveFML performs comparably well to other tested methods, and in some cohorts it outperforms all other methods. When its low rate of false positives (demonstrated by its unparalleled performance in the qq plots) and its satisfactory rate of true positives are taken together, OncodriveFML is revealed as a very powerful method for the detection of putative driver coding genes.

Finally, we compared the sets of putative driver genes identified by the latest release of MutSigCV, obtained from the Broad Institute Tumor Portal (see main manuscript for details) in four cohorts of tumors (Figure 5B, below). Because only significant genes could be retrieved from the Tumor Portal website, we only assessed the size of the overlap between the lists of putative drivers detected by both methods. Despite the fact that in the four cases presented below the overlap was clearly enriched for known cancer genes, some known and *bona fide* new drivers were detected by only one of the methods, emphasizing the importance of employing them in combination.

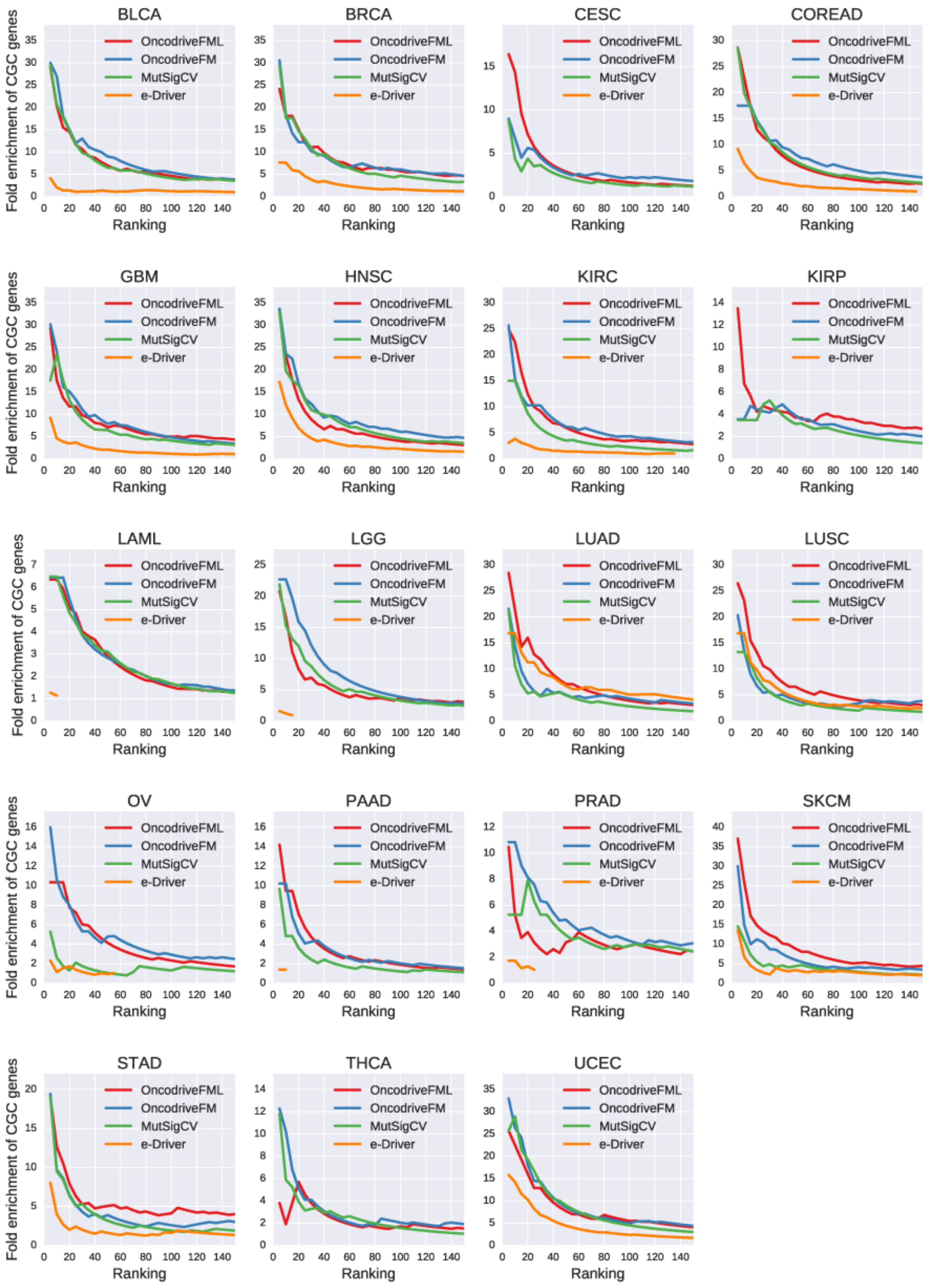

Figure 5A. Performance of five methods in the detection of putative driver genes from 19 datasets of mutations in coding genes in the same number of cohorts of tumors. The performance is assessed as the increase in the ratio of known cancer genes (from the CGC) each method retrieves within increasing numbers of their top-ranking genes.

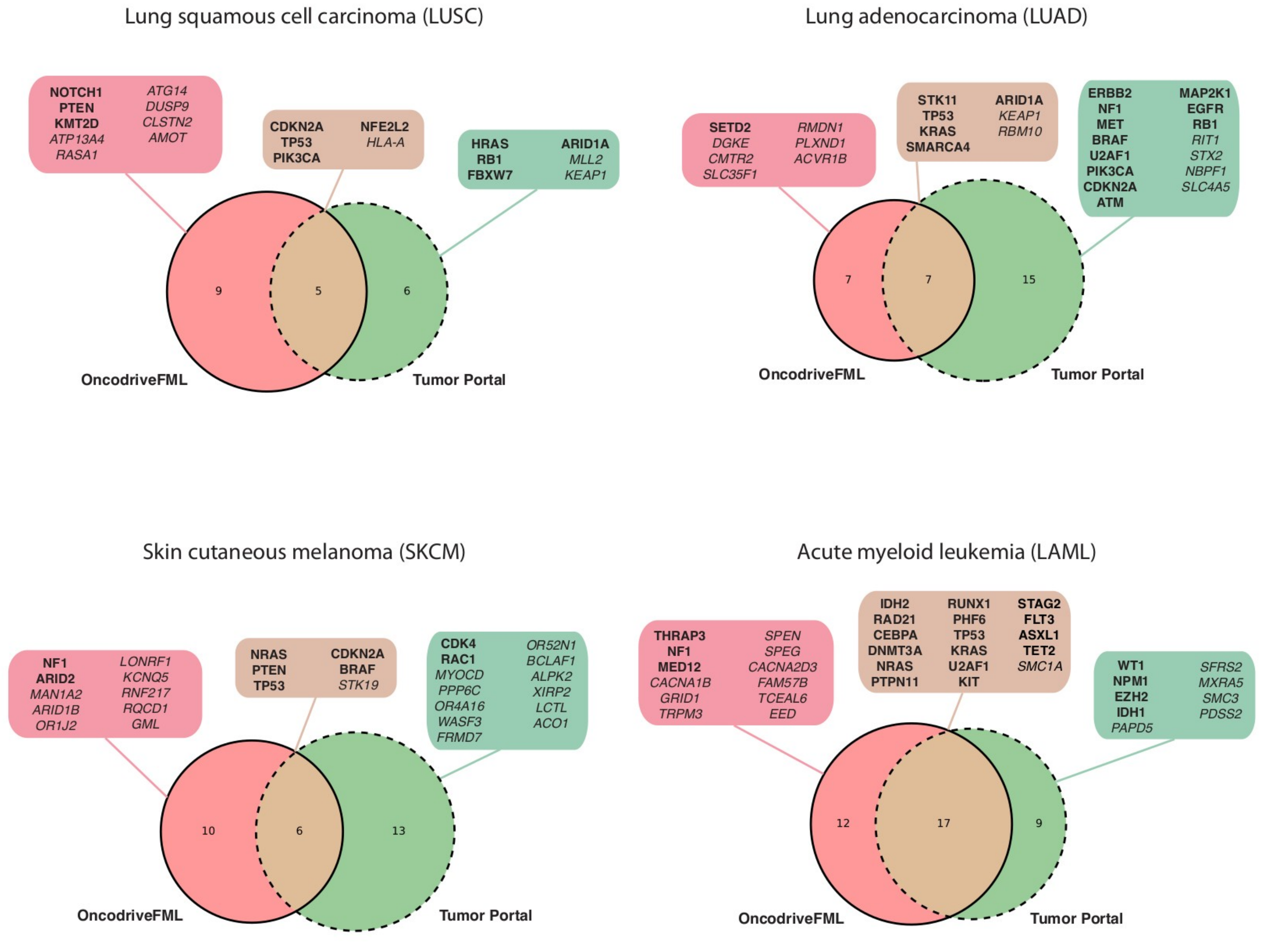

Figure 5B. Overlap between drivers detected by the latest release of MutSigCV obtained from the Broad Institute Tumor Portal (see main manuscript) and those identified by OncodriveFML on four cohorts of tumors. Known cancer genes (form the CGC) appear in bold.

B) Benchmark of the detection of driver non-coding elements

We have compared the results of OncodriveFML to those of two recently published and publicly available methods, SASE-hunter (Smith et al., 2015) and LARVA (Lochovsky, Zhang, Fu, Khurana, & Gerstein, 2015). We applied the three methods to both WG-505 and WG-608 whole-genome datasets, for the identification of potentially driver promoters, UTRs, and splice intronic regions. The first dataset contains the somatic mutations of 7 different cancer types for a total of 608 samples (WG-608). The second dataset contains the somatic mutations of 14 different cancer types for a total of 505 samples (WG-505). The sources of the data along with the number of samples and the number of mutations of each cancer type are listed in Additional data file 1. SASE-hunter was run setting the parameters '--upstream' and '--downstream' to 20000 and performing a fisher test. When the program was run on splice intronic regions we excluded from the analysis (with the parameter –exclude) any region defined as CDS. Similarly, when the program was run on 3' UTRs beside CDS we also excluded from the analysis short splice intronic regions. In 5' UTRs we excluded CDS, short splice intronic regions, and 3' UTRs, In promoters we applied the same filters as in 5' UTRs and in addition we excluded 5' UTRs. Only elements with at least one mutated sample were included in the comparison. LARVA was run using the docker image provided by the authors. Only elements with at least one mutation were included in the comparison.

Because no CGC-like gold standard of driver non-coding elements exists beyond scattered reports in the literature of recurrently mutated features – such as the promoter of TERT –, we only benchmarked OncodriveFML with respect to the aforementioned methods through the distribution of their observed p-values via qq-plots. In figures 6, 7, and 8 we present the distributions of p-values of SASE-hunter, LARVA, and OncodriveFML, respectively in promoters on the WG-505 dataset. Analogous graphs for the distribution of p-values of 3' UTRs, 5' UTRs, and splice intronic regions are depicted in figures 9-17. Figures 18-29 present the results obtained on cohorts of the WG-608 dataset.

These figures clearly show that OncodriveFML produces a well calibrated distribution of p-values that follows the null hypothesis with the exception of few significant driver candidates in the case of all genomic elements analyzed. On the other hand, both SASE-hunter and LARVA often show a rate of very low p-values much greater than the expected distribution of the null hypothesis. These inflated p-values may lead to a great rate of false positive in the analysis.

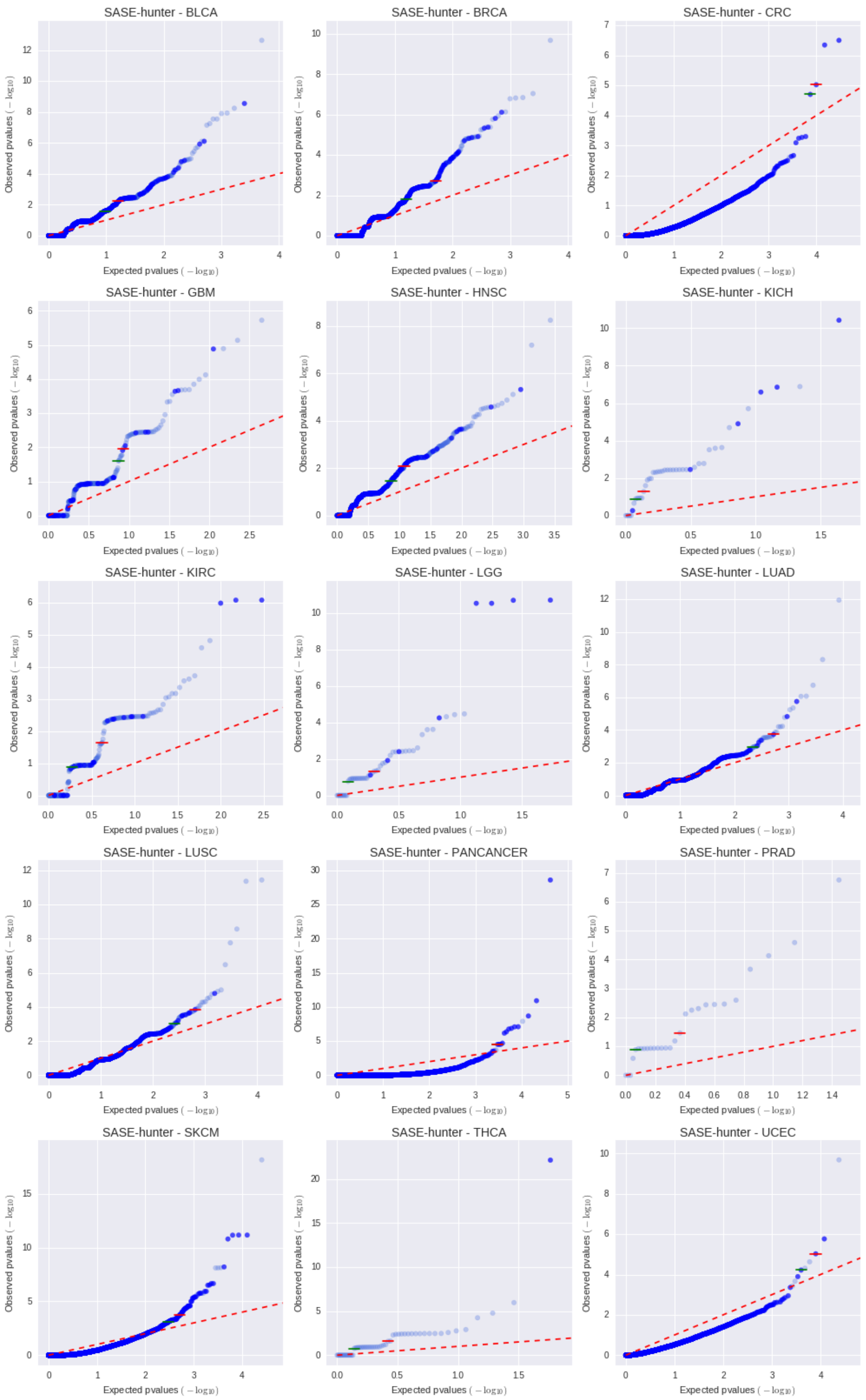

Figure 6. Quantile-quantile (qq) plots comparing the distribution of observed and expected p-values of tests carried out by SASE-hunter on mutations in promoter regions in 14 cohorts of tumors (from dataset WG-505) and the corresponding pan-cancer cohort. False discovery rate thresholds (0.1 and 0.25) are indicated as short red and green segments in each graph.

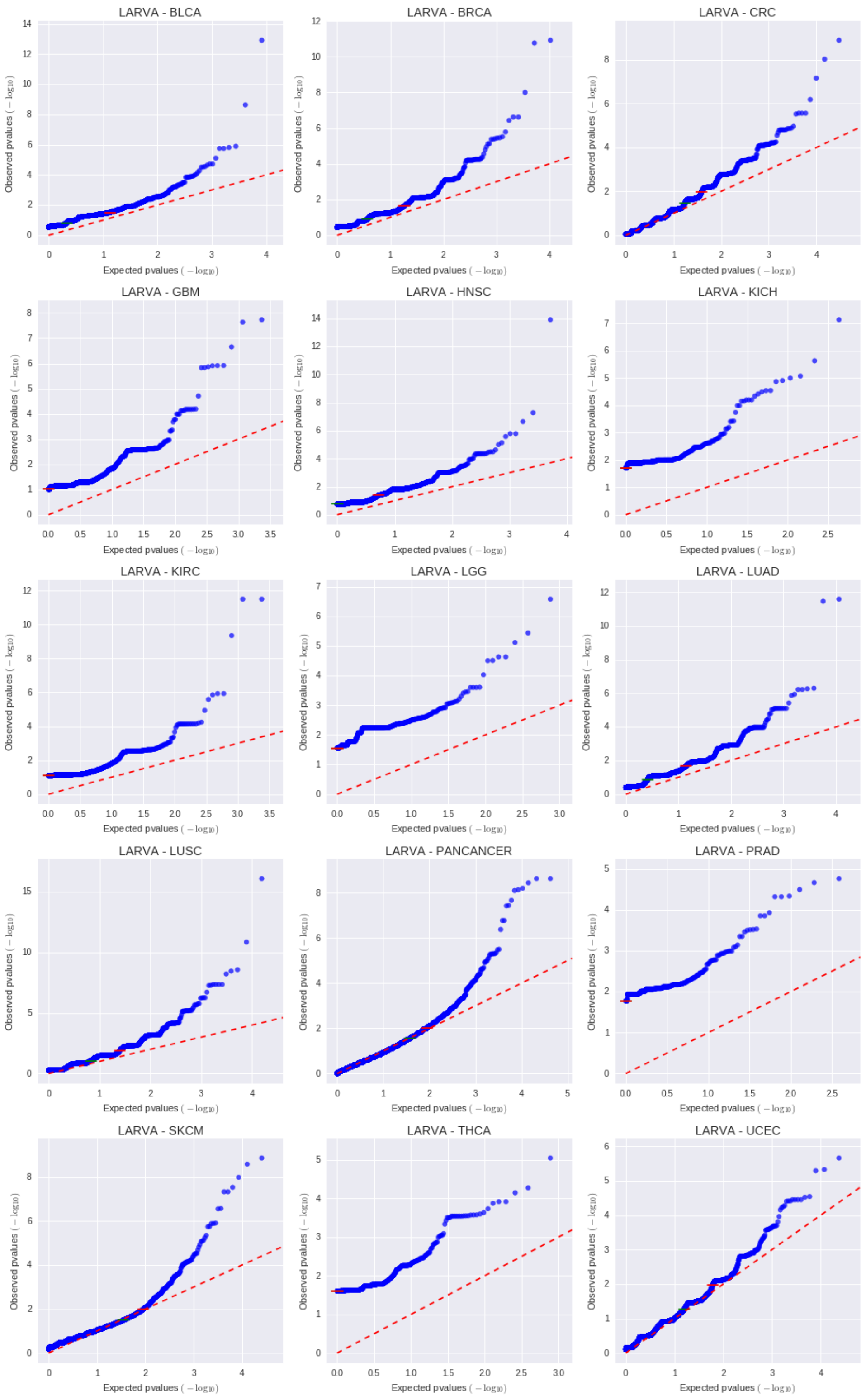

Figure 7. Quantile-quantile (qq) plots comparing the distribution of observed and expected p-values of tests carried out by LARVA on mutations in promoter regions in 14 cohorts of tumors (from dataset WG-505) and the corresponding pan-cancer cohort. False discovery rate thresholds (0.1 and 0.25) are indicated as short red and green segments in each graph.

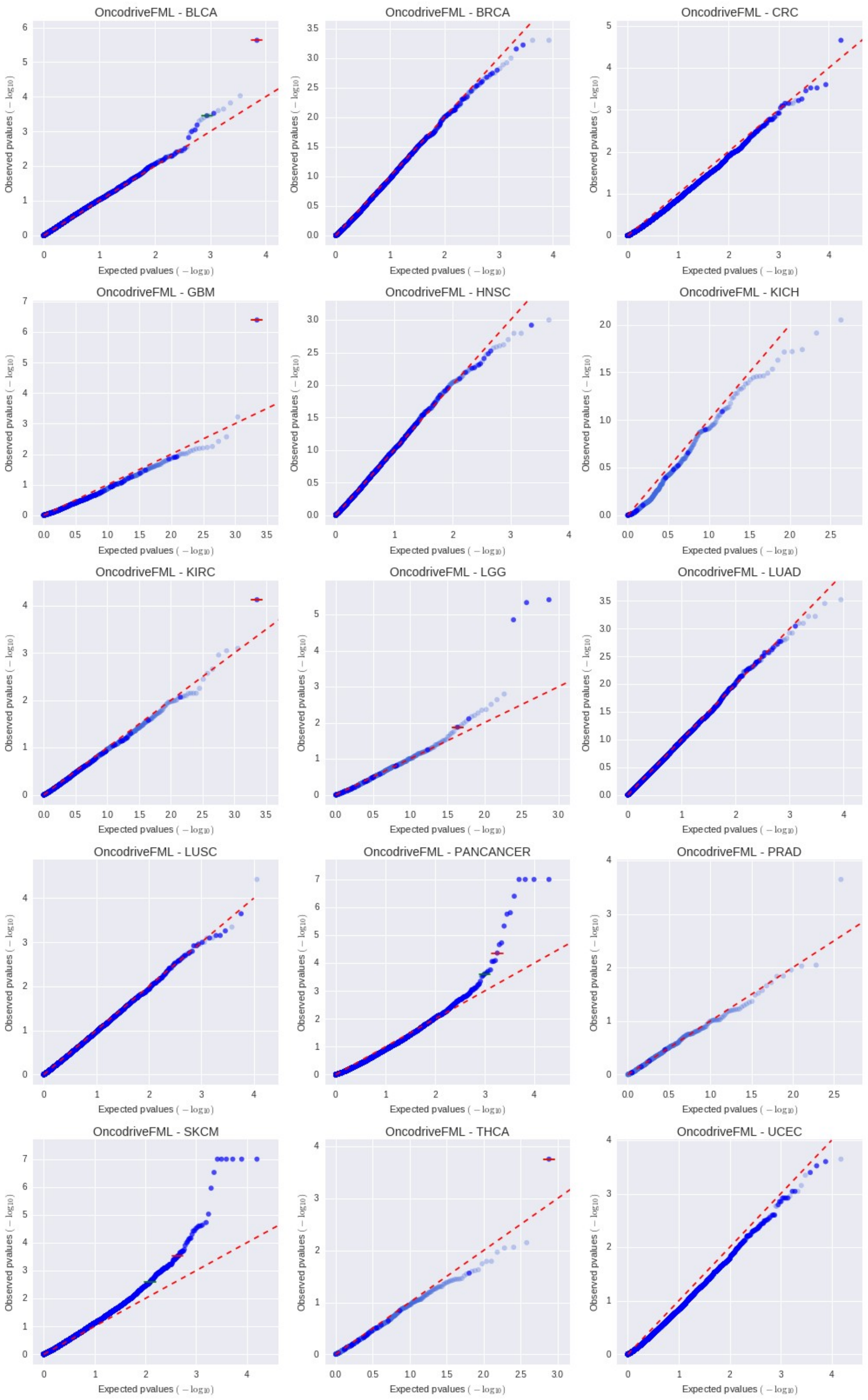

Figure 8. Quantile-quantile (qq) plots comparing the distribution of observed and expected p-values of tests carried out by OncodriveFML on mutations in promoter regions in 14 cohorts of tumors (from dataset WG-505) and the corresponding pan-cancer cohort. False discovery rate thresholds (0.1 and 0.25) are indicated as short red and green segments in each graph.

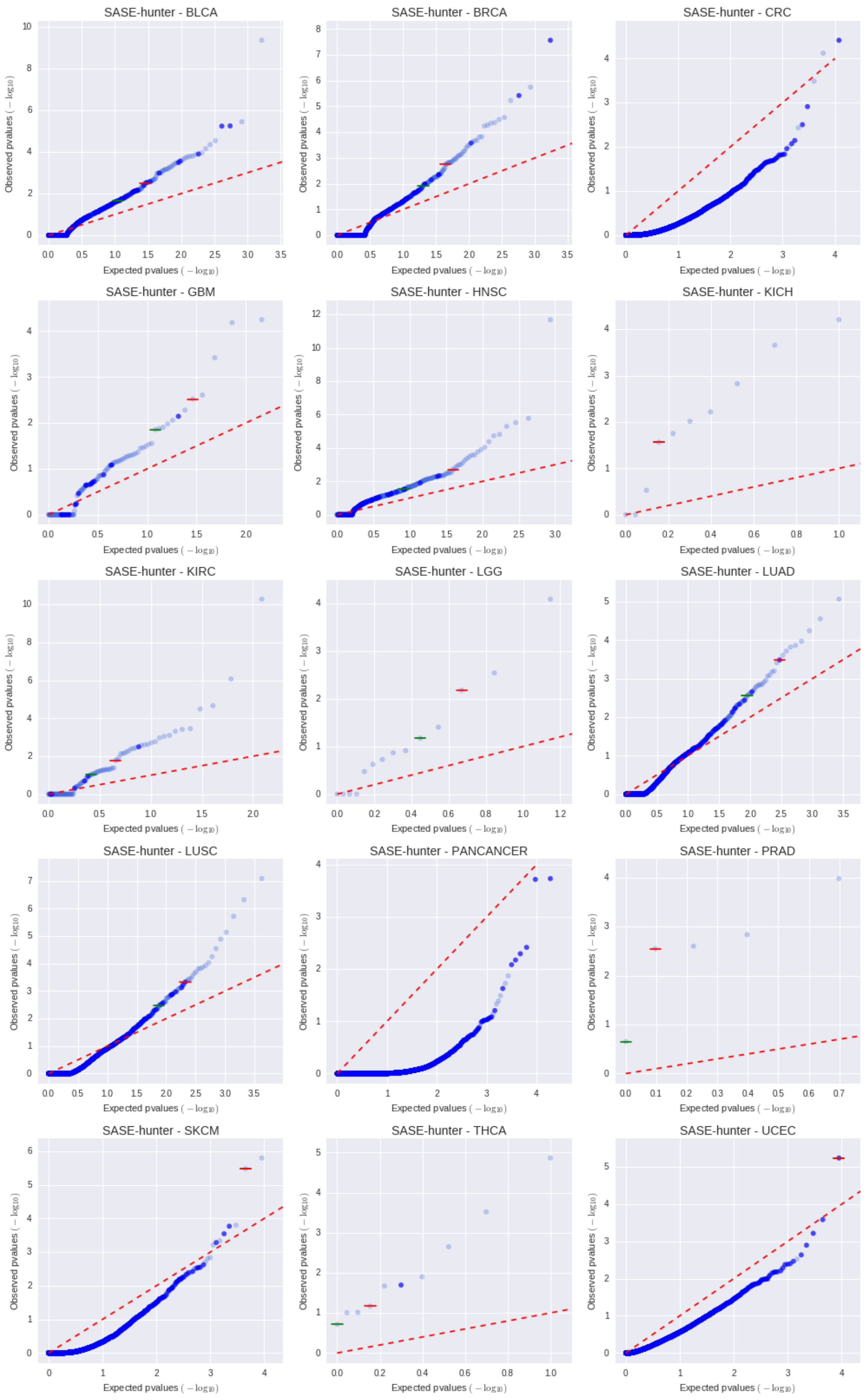

Figure 9. Quantile-quantile (qq) plots comparing the distribution of observed and expected p-values of tests carried out by SASE-hunter on mutations in 3' UTRs in 14 cohorts of tumors (from dataset WG-505) and the corresponding pan-cancer cohort. False discovery rate thresholds (0.1 and 0.25) are indicated as short red and green segments in each graph.

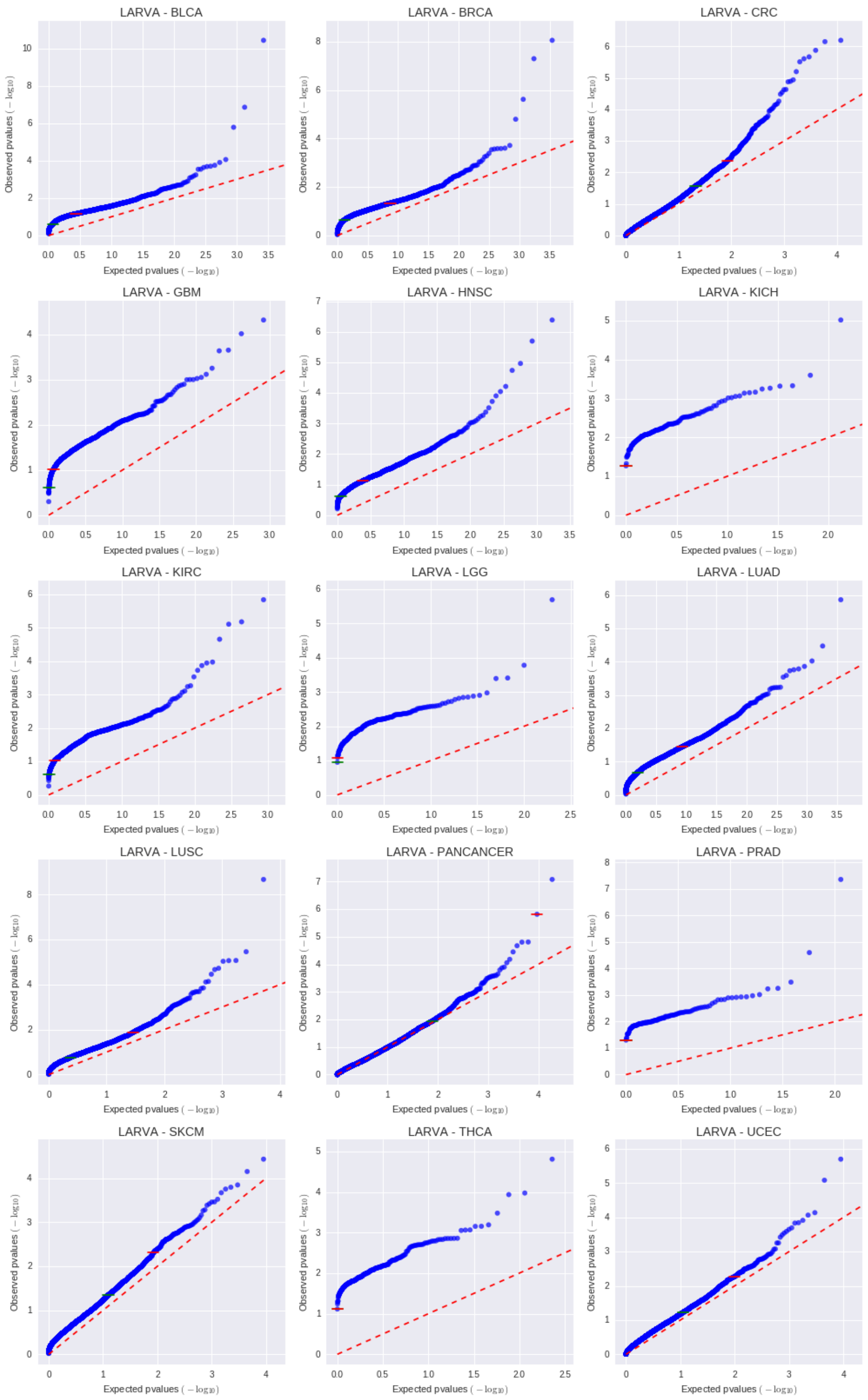

Figure 10. Quantile-quantile (qq) plots comparing the distribution of observed and expected p-values of tests carried out by LARVA on mutations in 3' UTRs in 14 cohorts of tumors (from dataset WG-505) and the corresponding pan-cancer cohort. False discovery rate thresholds (0.1 and 0.25) are indicated as short red and green segments in each graph.

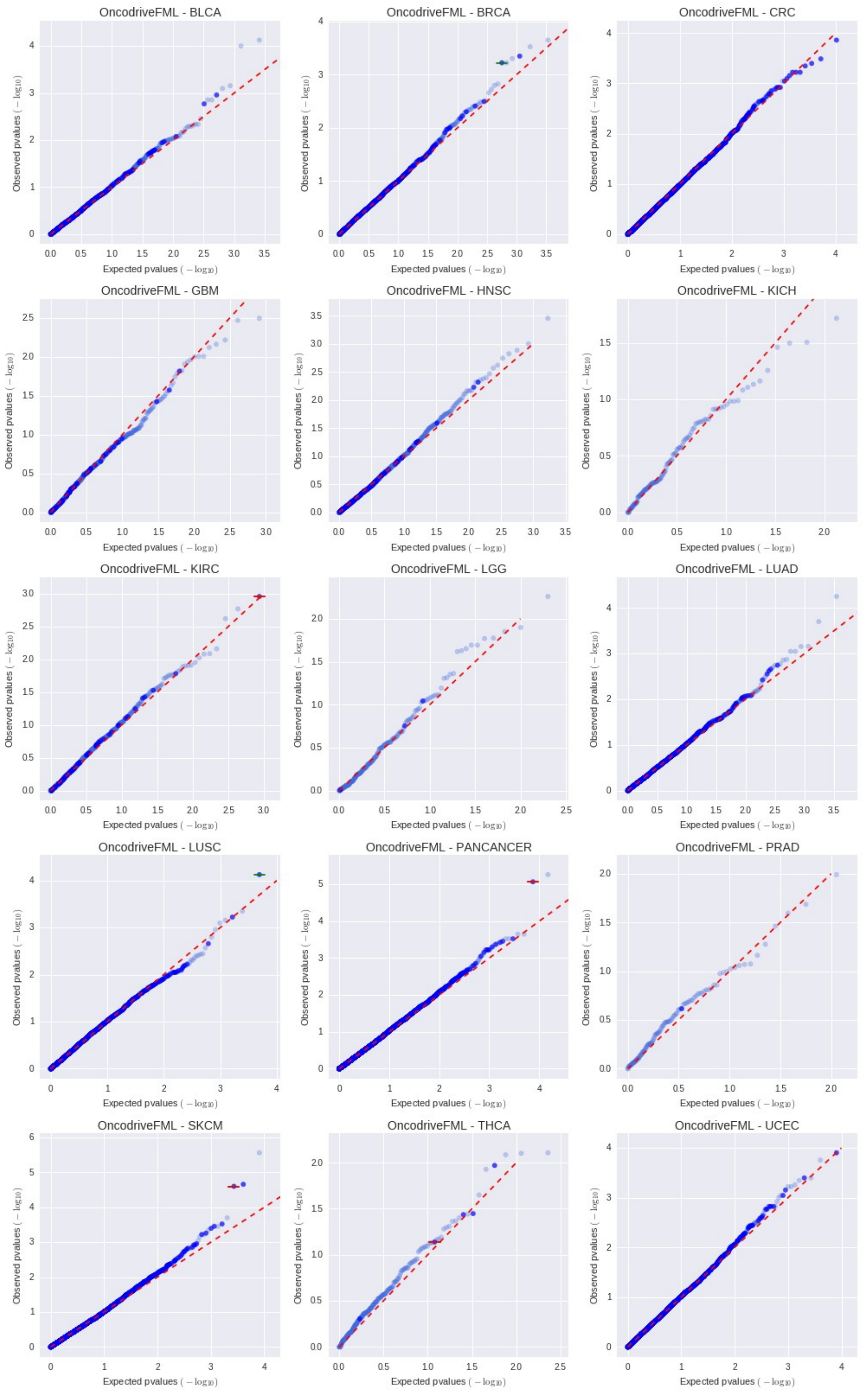

Figure 11. Quantile-quantile (qq) plots comparing the distribution of observed and expected p-values of tests carried out by OncodriveFML on mutations in 3' UTRs in 14 cohorts of tumors (from dataset WG-505) and the corresponding pan-cancer cohort. False discovery rate thresholds (0.1 and 0.25) are indicated as short red and green segments in each graph.

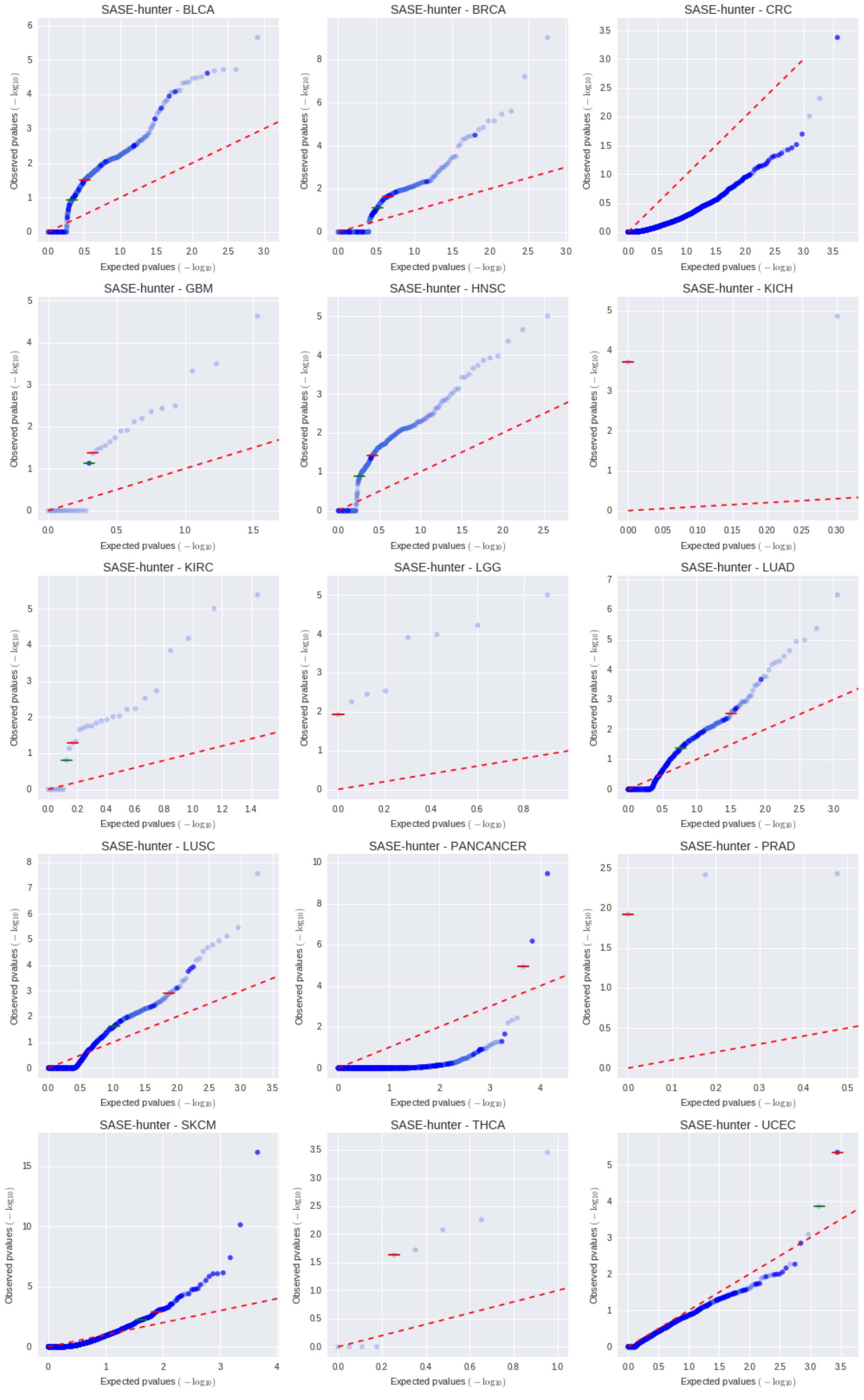

Figure 12. Quantile-quantile (qq) plots comparing the distribution of observed and expected p-values of tests carried out by SASE-hunter on mutations in 5' UTRs in 14 cohorts of tumors (from dataset WG-505) and the corresponding pan-cancer cohort. False discovery rate thresholds (0.1 and 0.25) are indicated as short red and green segments in each graph.

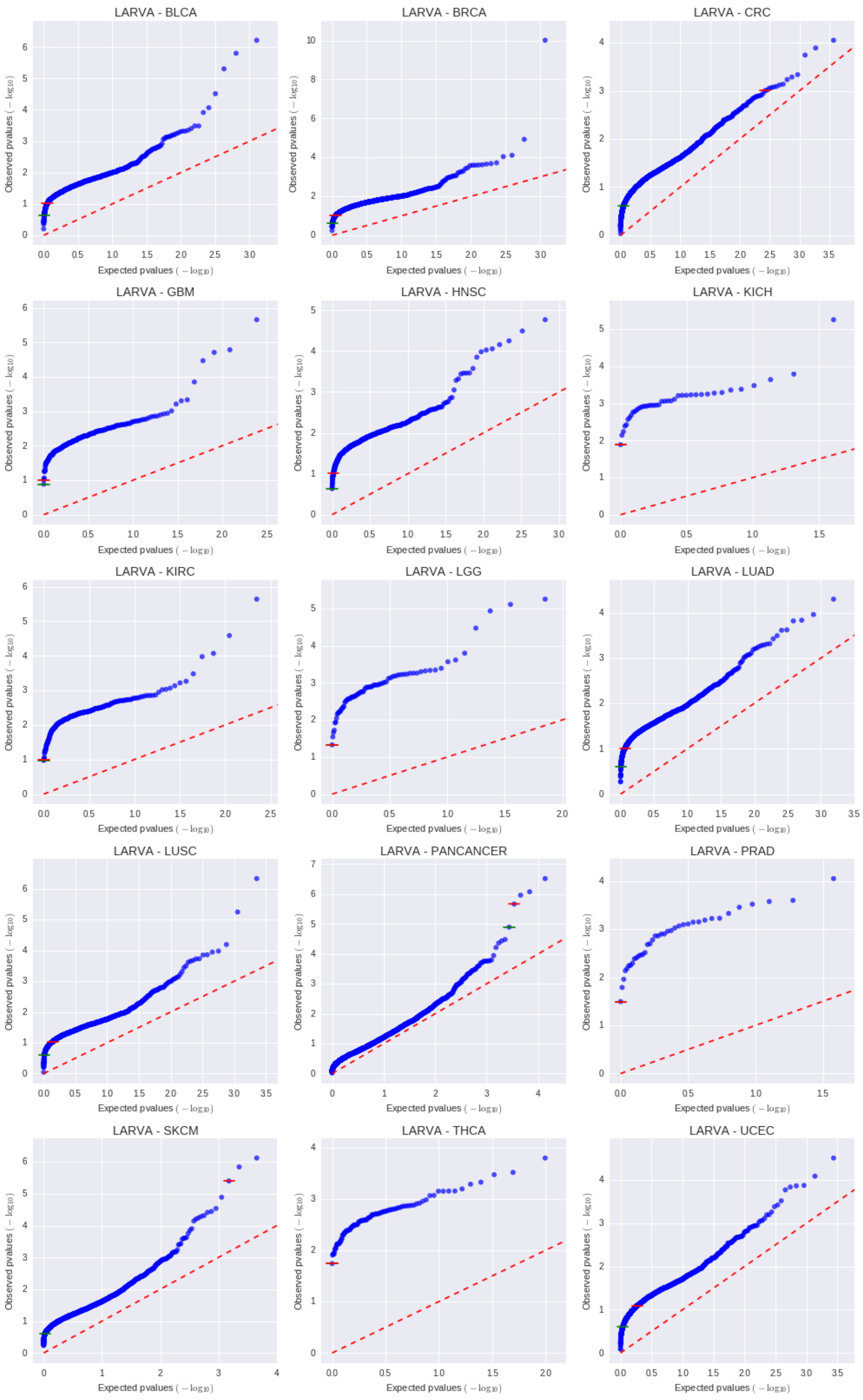

Figure 13. Quantile-quantile (qq) plots comparing the distribution of observed and expected p-values of tests carried out by LARVA on mutations in 5' UTRs in 14 cohorts of tumors (from dataset WG-505) and the corresponding pan-cancer cohort. False discovery rate thresholds (0.1 and 0.25) are indicated as short red and green segments in each graph.

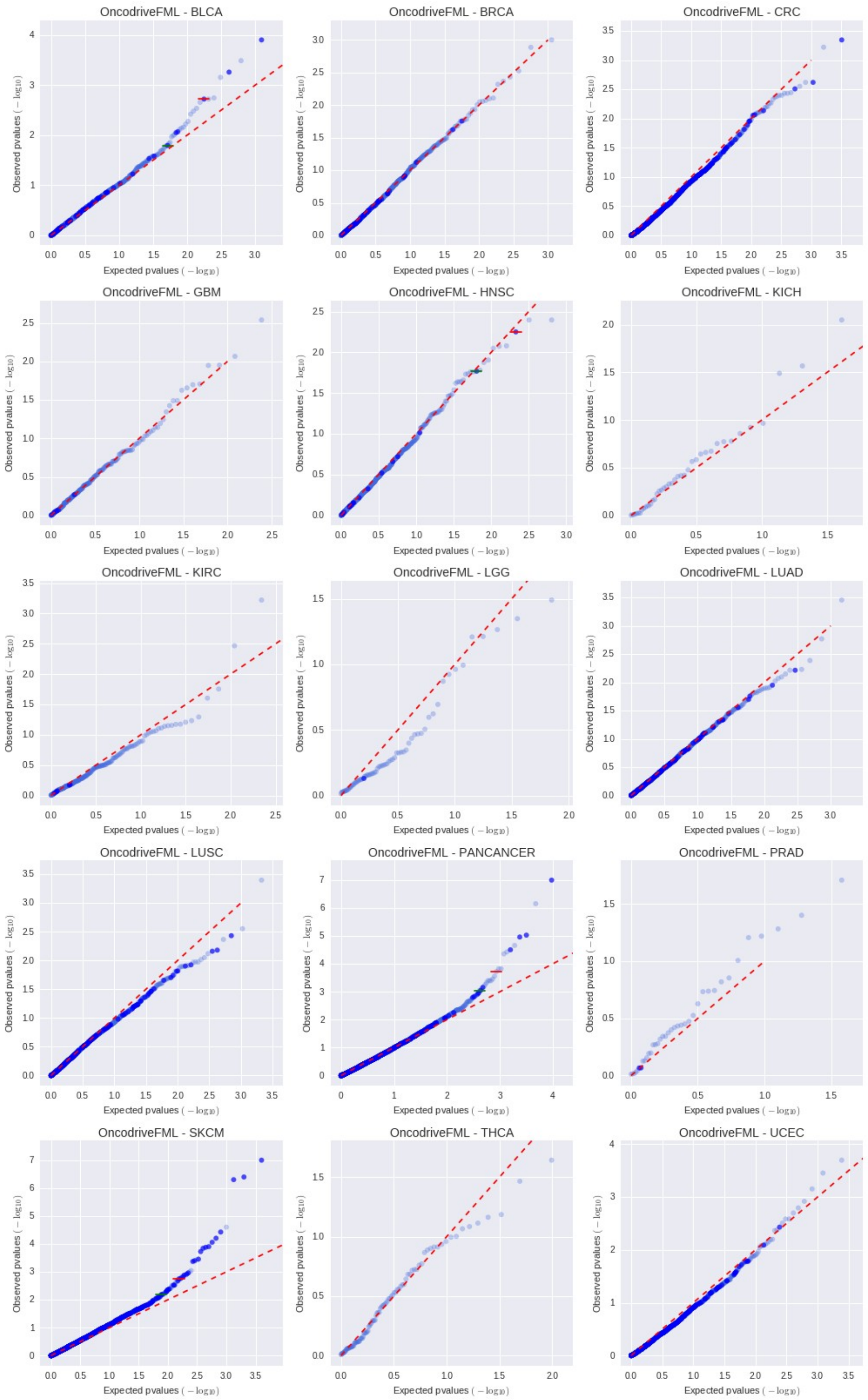

Figure 14. Quantile-quantile (qq) plots comparing the distribution of observed and expected p-values of tests carried out by OncodriveFML on mutations in 5' UTRs in 14 cohorts of tumors (from dataset WG-505) and the corresponding pan-cancer cohort. False discovery rate thresholds (0.1 and 0.25) are indicated as short red and green segments in each graph.

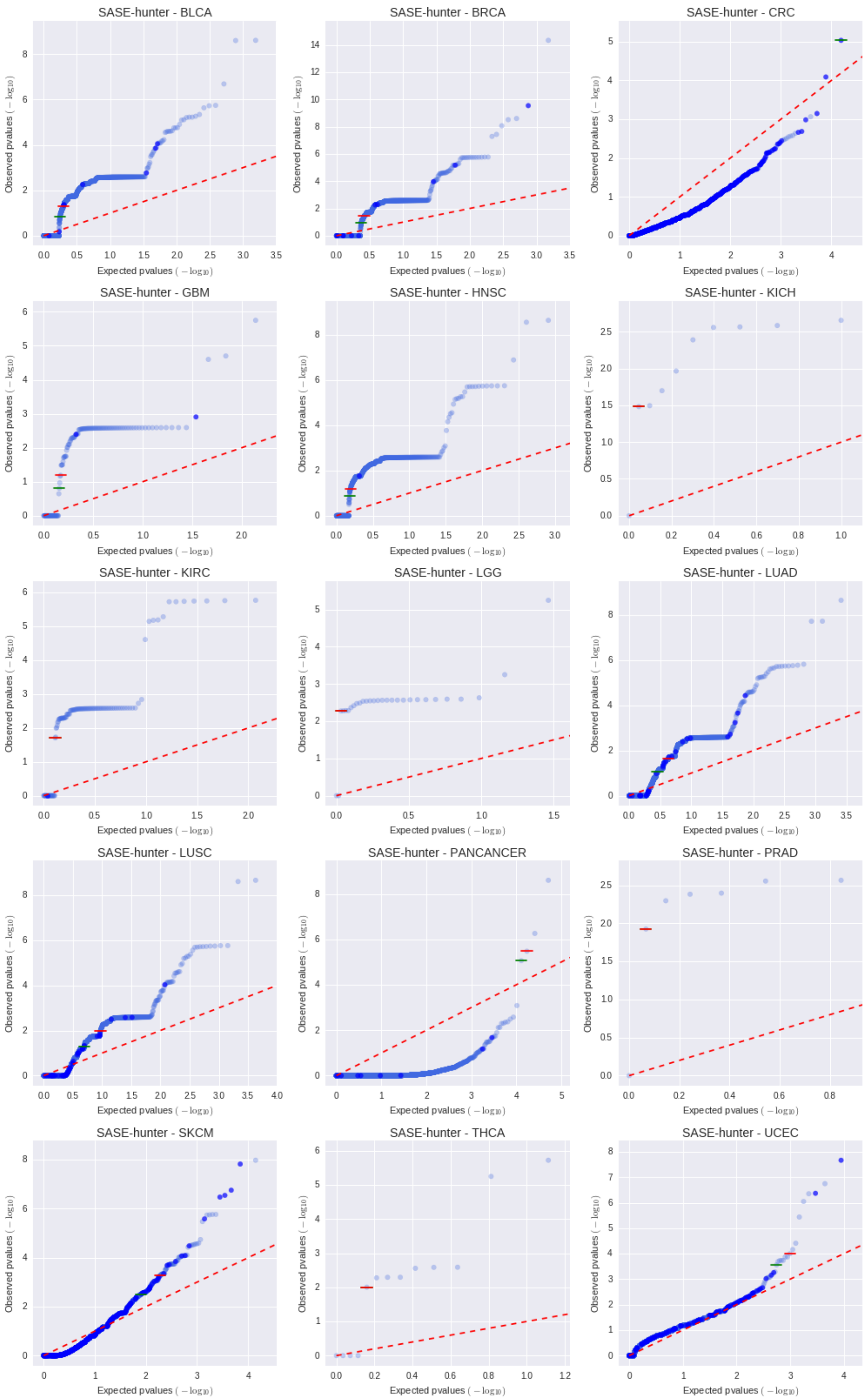

Figure 15. Quantile-quantile (qq) plots comparing the distribution of observed and expected p-values of tests carried out by SASE-hunter on mutations in splice intronic regions in 14 cohorts of tumors (from dataset WG-505) and the corresponding pan-cancer cohort. False discovery rate thresholds (0.1 and 0.25) are indicated as short red and green segments in each graph.

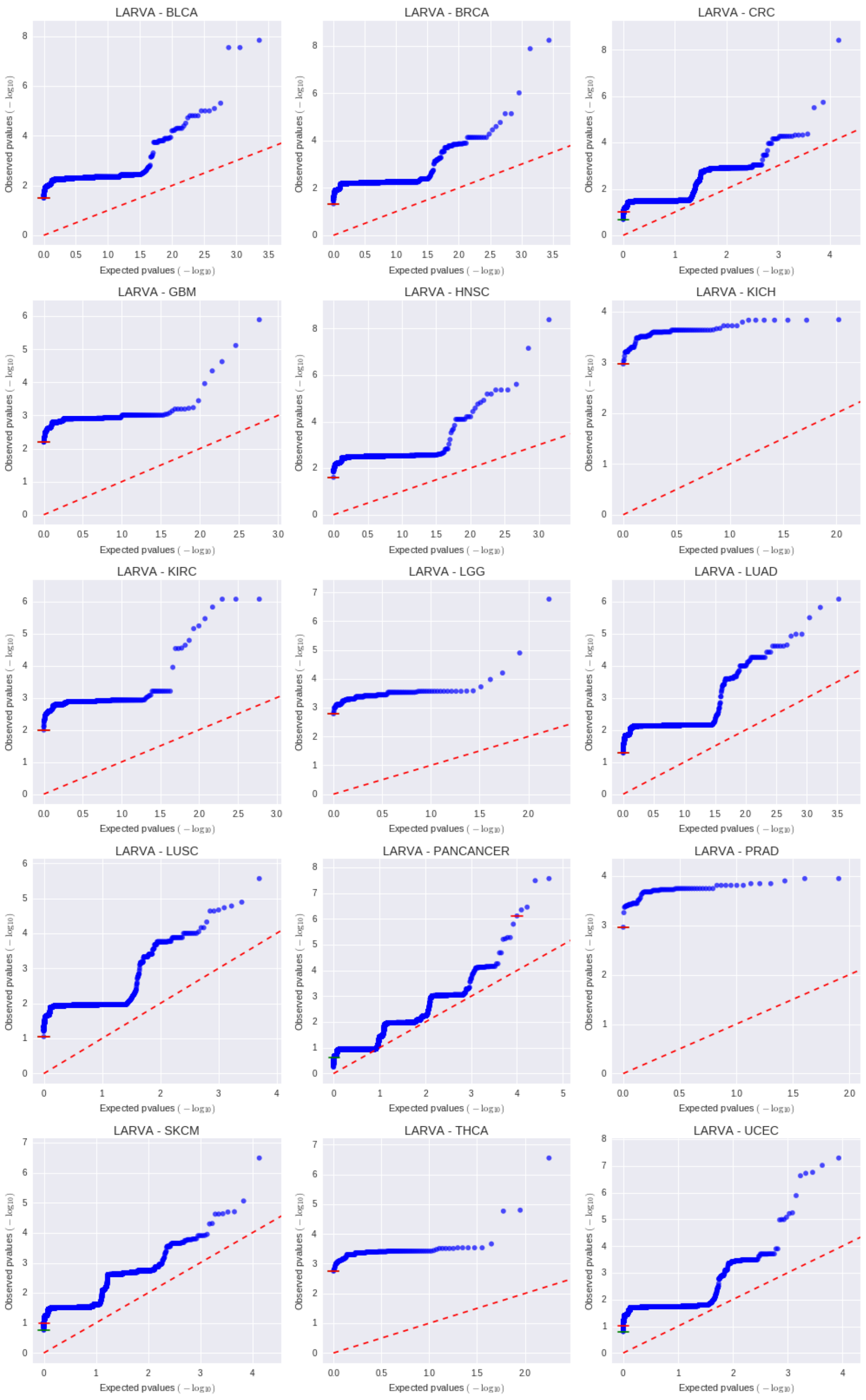

Figure 16. Quantile-quantile (qq) plots comparing the distribution of observed and expected p-values of tests carried out by LARVA on mutations in splice intronic regions regions in 14 cohorts of tumors (from dataset WG-505) and the corresponding pan-cancer cohort. False discovery rate thresholds (0.1 and 0.25) are indicated as short red and green segments in each graph.

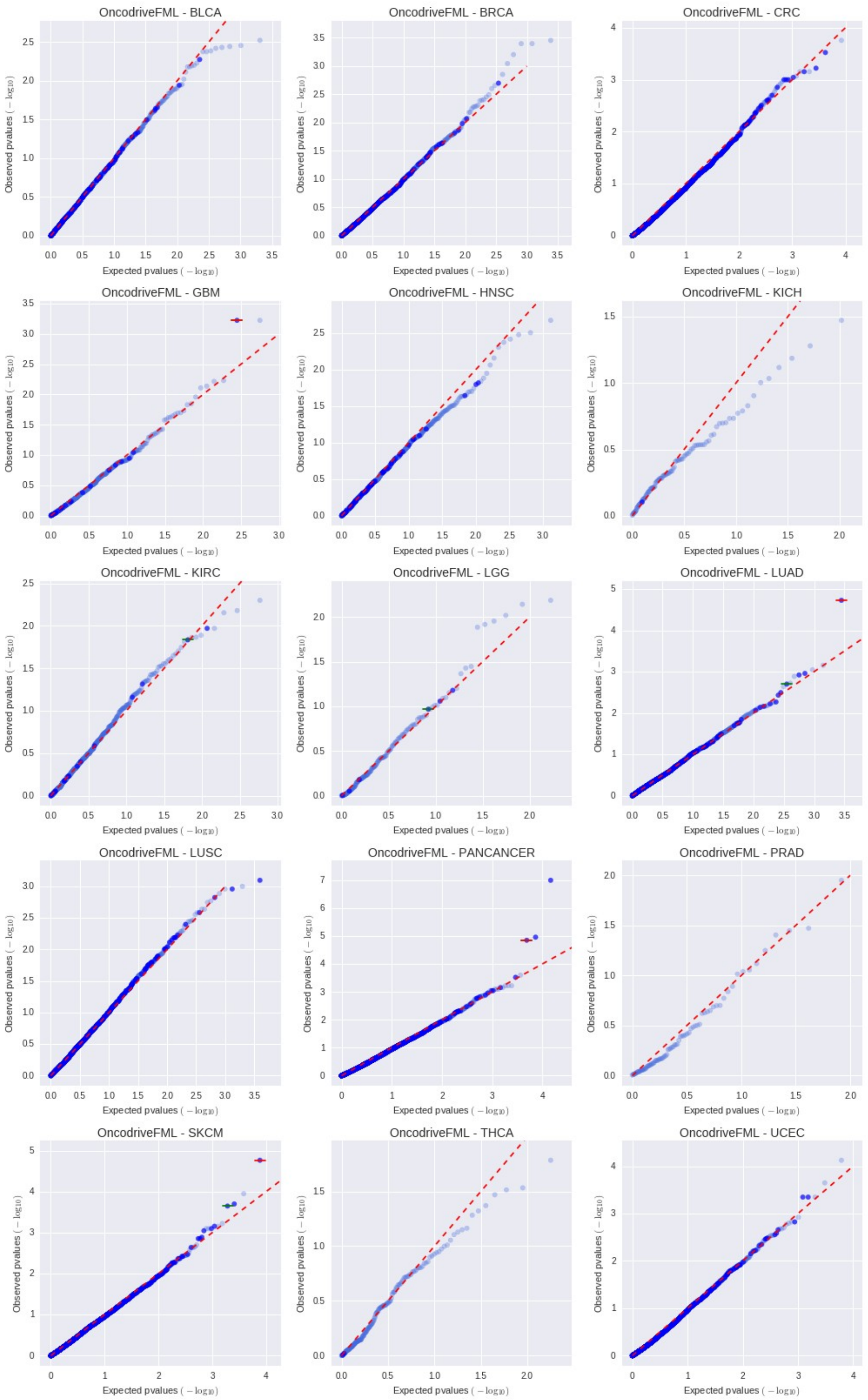

Figure 17. Quantile-quantile (qq) plots comparing the distribution of observed and expected p-values of tests carried out by OncodriveFML on mutations in splice intronic regions regions in 14 cohorts of tumors (from dataset WG-505) and the corresponding pan-cancer cohort. False discovery rate thresholds (0.1 and 0.25) are indicated as short red and green segments in each graph.

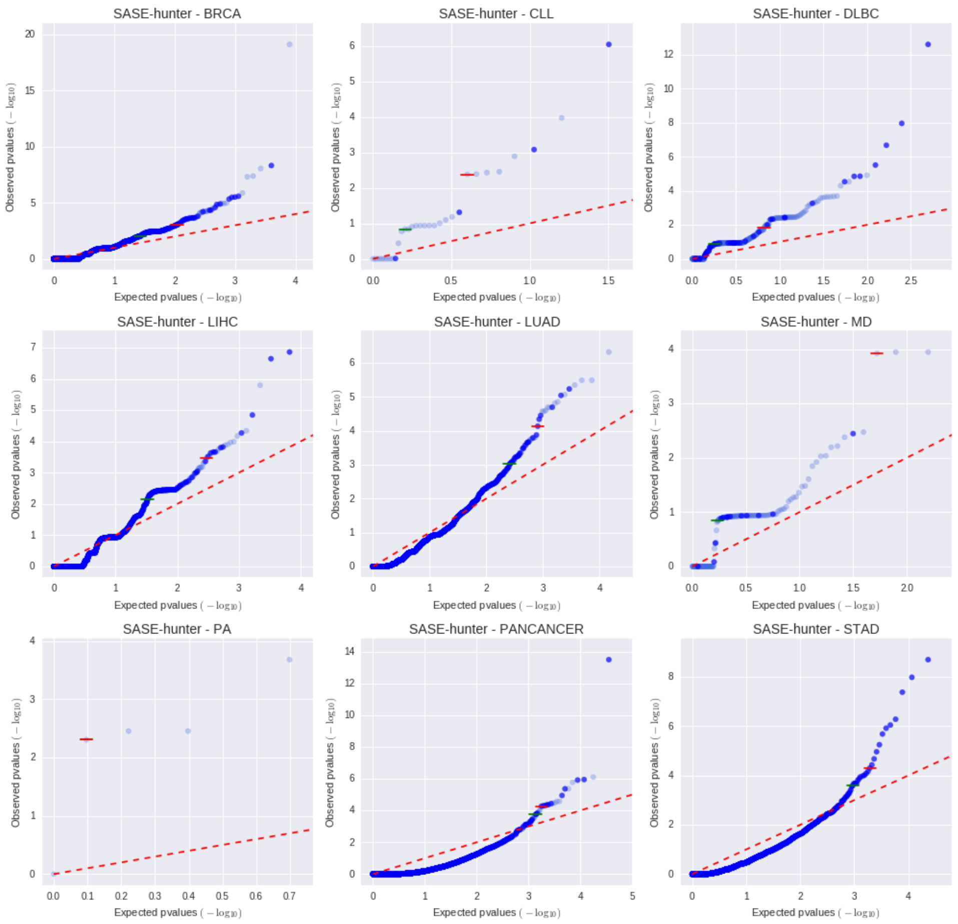

Figure 18. Quantile-quantile (qq) plots comparing the distribution of observed and expected p-values of tests carried out by SASE-hunter on mutations in promoter regions in 8 cohorts of tumors (from dataset WG-608) and the corresponding pan-cancer cohort. False discovery rate thresholds (0.1 and 0.25) are indicated as short red and green segments in each graph.

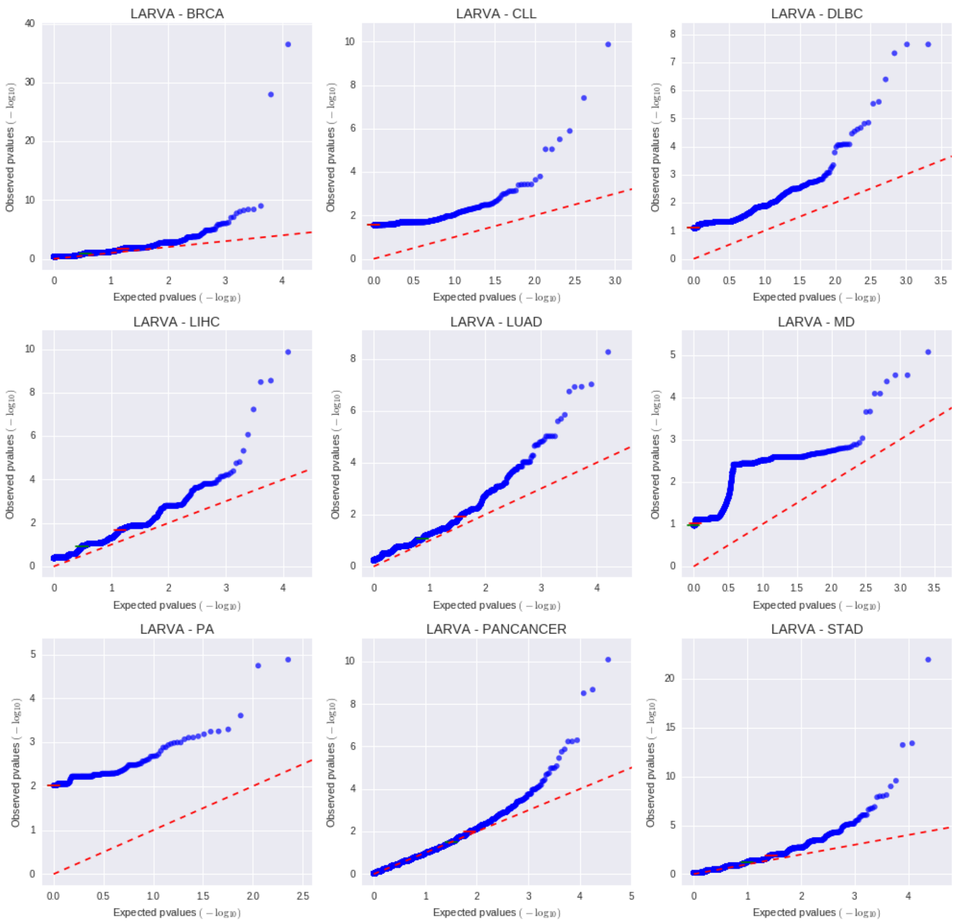

Figure 19. Quantile-quantile (qq) plots comparing the distribution of observed and expected p-values of tests carried out by LARVA on mutations in promoter regions in 8 cohorts of tumors (from dataset WG-608) and the corresponding pan-cancer cohort. False discovery rate thresholds (0.1 and 0.25) are indicated as short red and green segments in each graph.

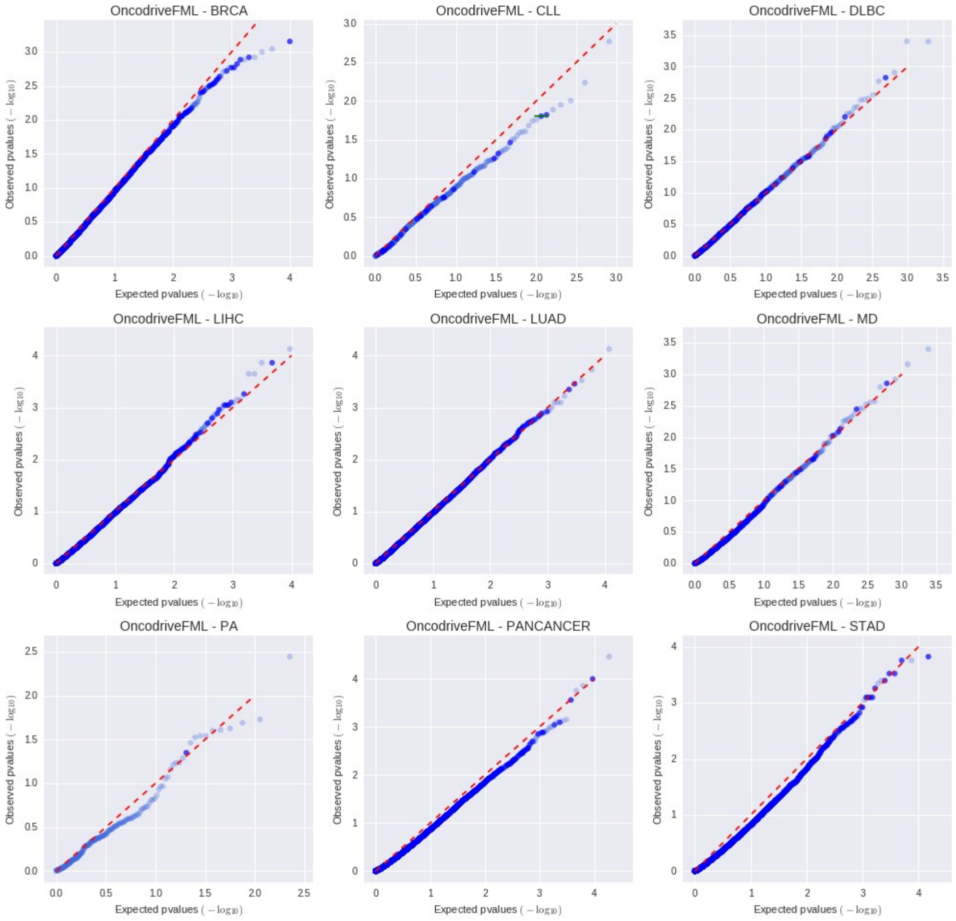

Figure 20. Quantile-quantile (qq) plots comparing the distribution of observed and expected p-values of tests carried out by OncodriveFML on mutations in promoter regions in 8 cohorts of tumors (from dataset WG-608) and the corresponding pan-cancer cohort. False discovery rate thresholds (0.1 and 0.25) are indicated as short red and green segments in each graph.

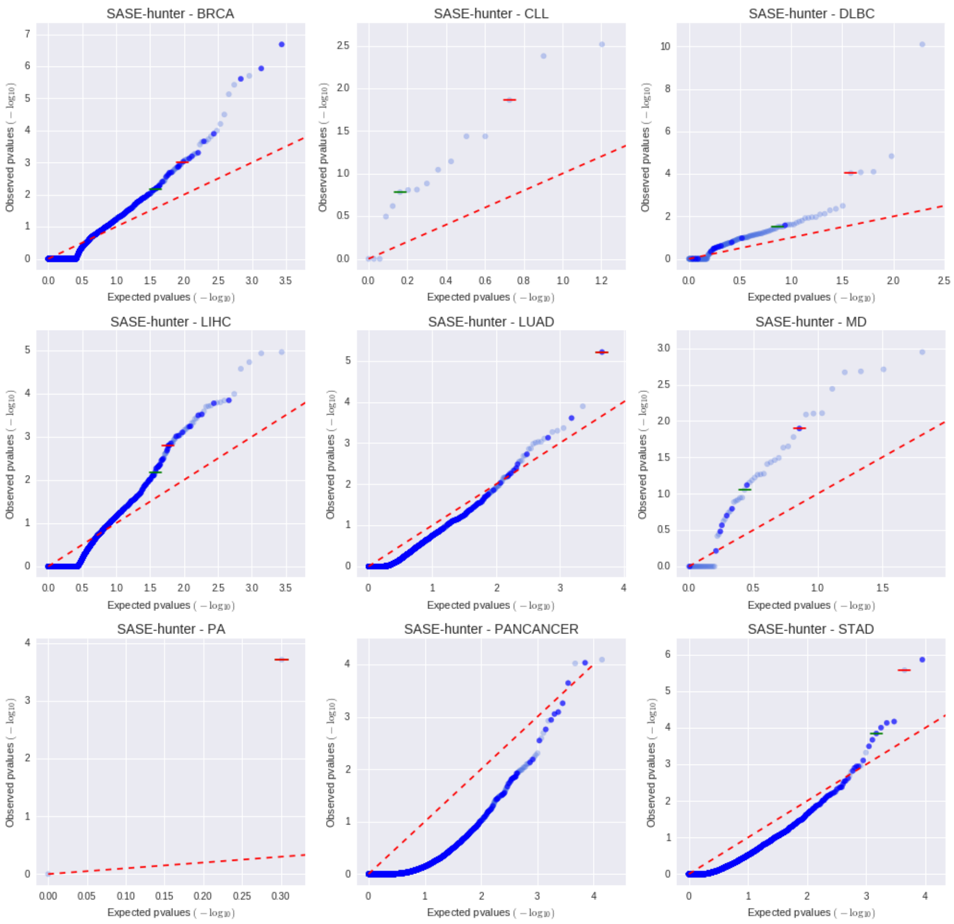

Figure 21. Quantile-quantile (qq) plots comparing the distribution of observed and expected p-values of tests carried out by SASE-hunter on mutations in 3' UTRs in 8 cohorts of tumors (from dataset WG-608) and the corresponding pan-cancer cohort. False discovery rate thresholds (0.1 and 0.25) are indicated as short red and green segments in each graph.

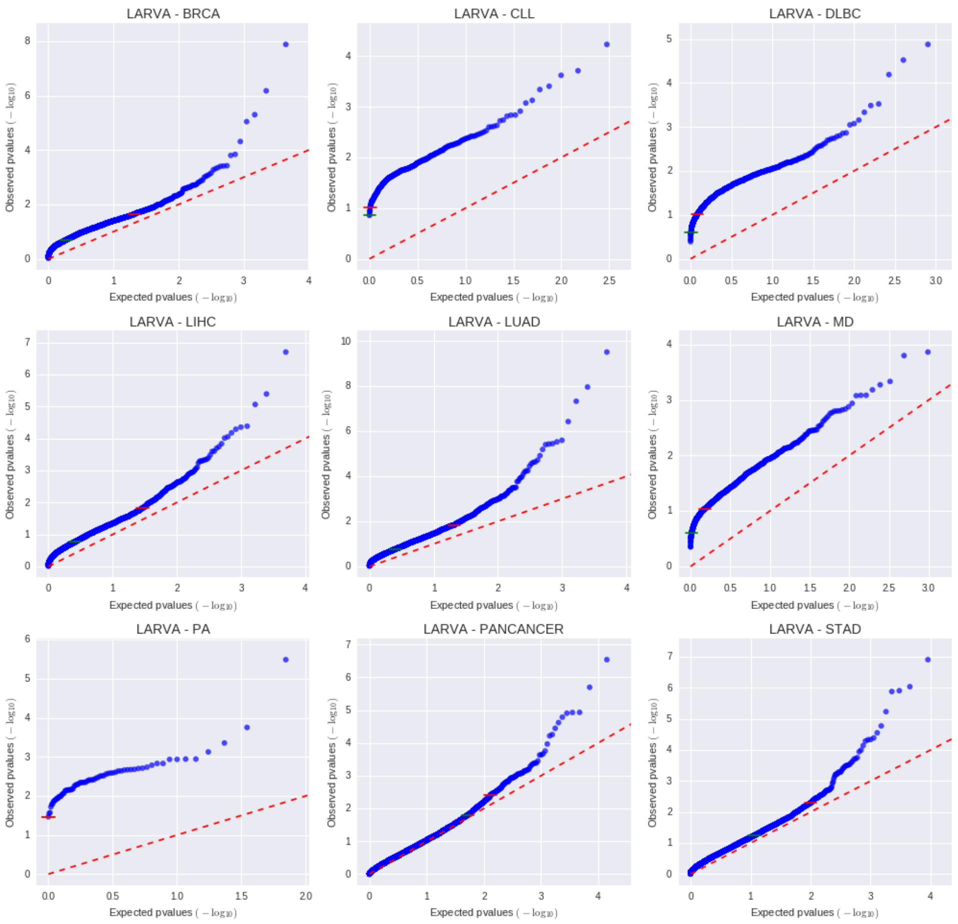

Figure 22. Quantile-quantile (qq) plots comparing the distribution of observed and expected p-values of tests carried out by LARVA on mutations in 3' UTRs in 8 cohorts of tumors (from dataset WG-608) and the corresponding pan-cancer cohort. False discovery rate thresholds (0.1 and 0.25) are indicated as short red and green segments in each graph.

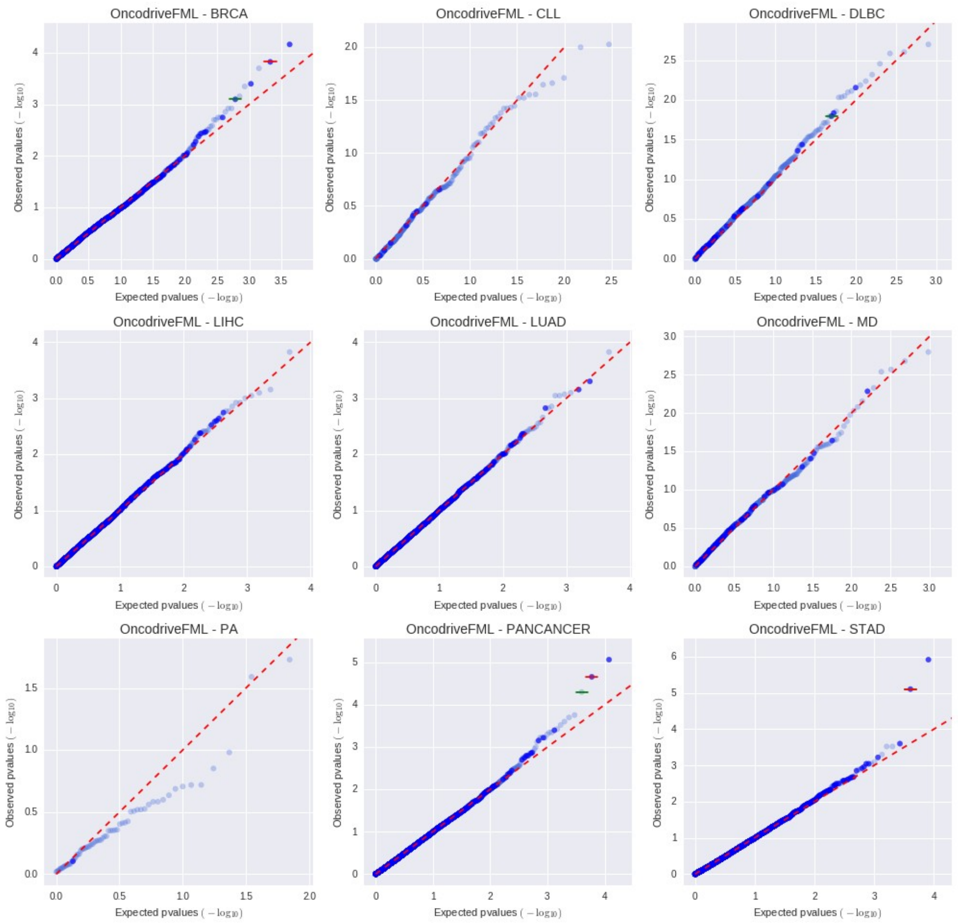

Figure 23. Quantile-quantile (qq) plots comparing the distribution of observed and expected p-values of tests carried out by OncodriveFML on mutations in 3' UTRs in 8 cohorts of tumors (from dataset WG-608) and the corresponding pan-cancer cohort. False discovery rate thresholds (0.1 and 0.25) are indicated as short red and green segments in each graph.

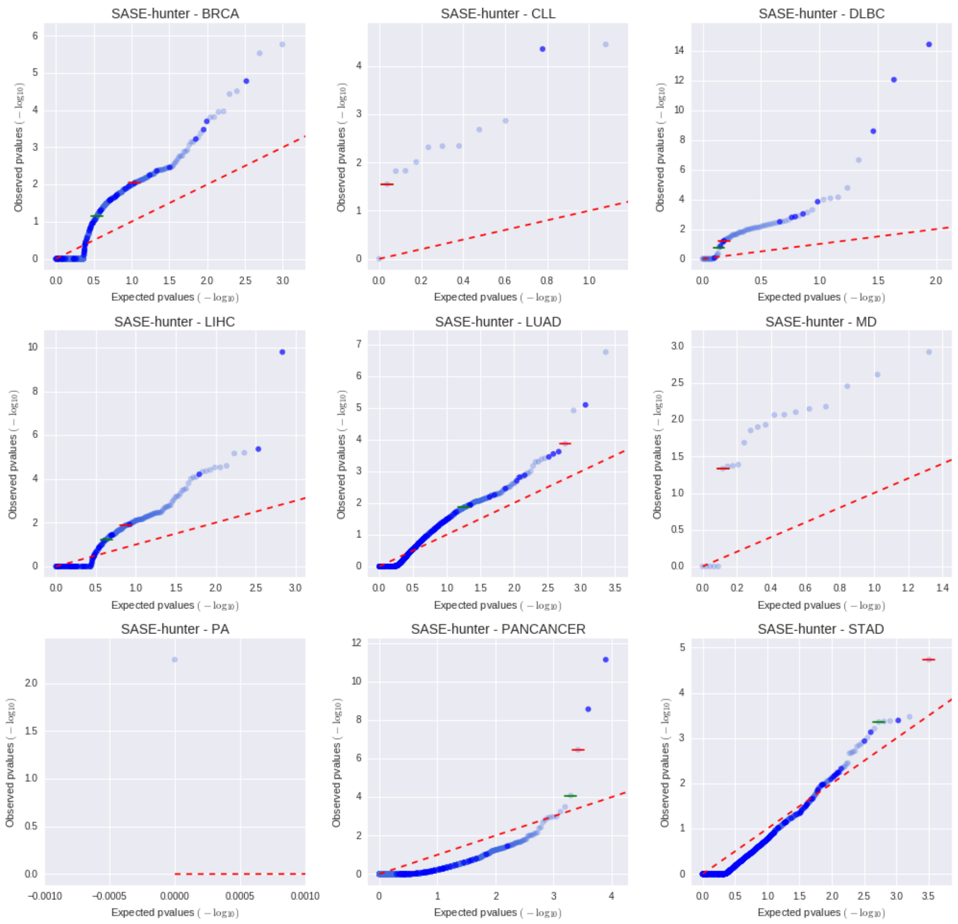

Figure 24. Quantile-quantile (qq) plots comparing the distribution of observed and expected p-values of tests carried out by SASE-hunter on mutations in 5' UTRs in 8 cohorts of tumors (from dataset WG-608) and the corresponding pan-cancer cohort. False discovery rate thresholds (0.1 and 0.25) are indicated as short red and green segments in each graph.

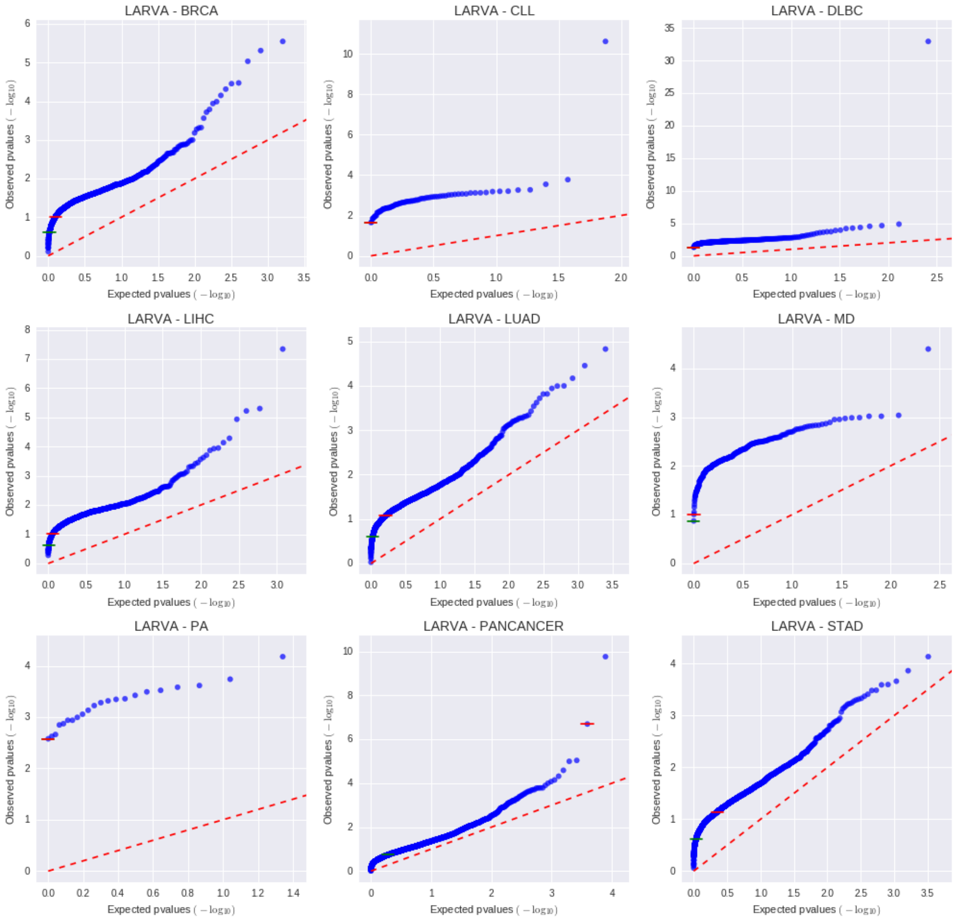

Figure 25. Quantile-quantile (qq) plots comparing the distribution of observed and expected p-values of tests carried out by LARVA on mutations in 5' UTRs in 8 cohorts of tumors (from dataset WG-608) and the corresponding pan-cancer cohort. False discovery rate thresholds (0.1 and 0.25) are indicated as short red and green segments in each graph.

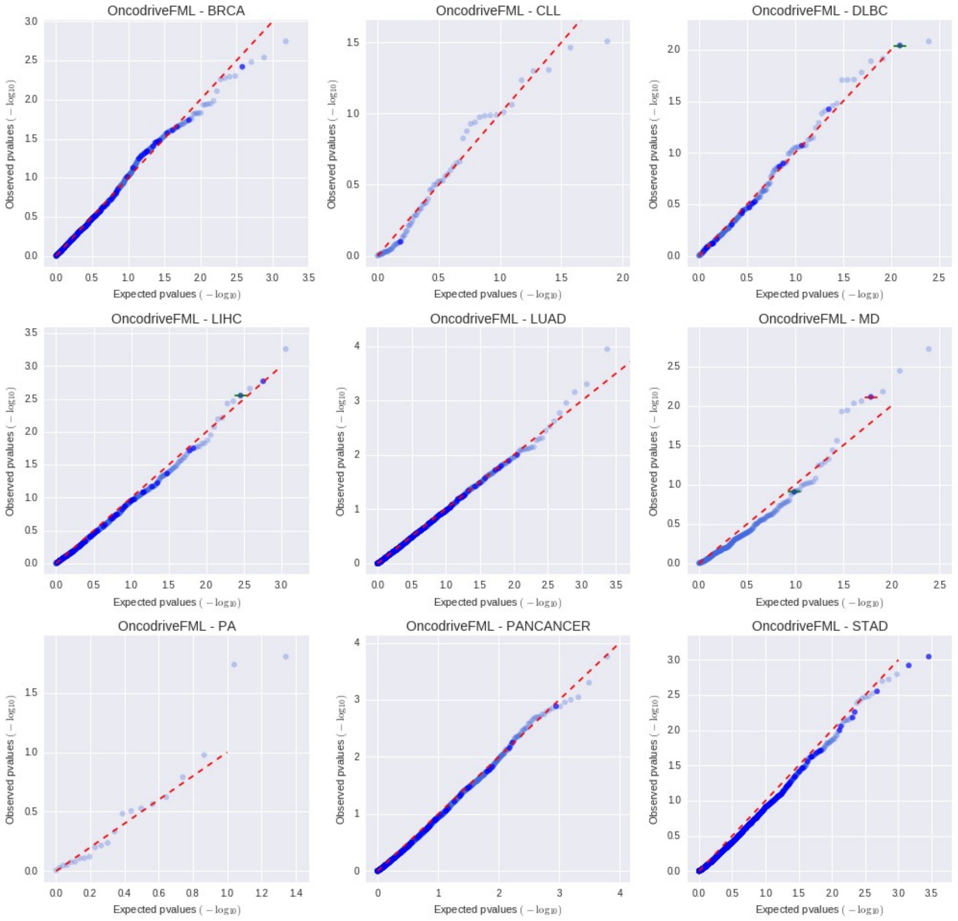

Figure 26. Quantile-quantile (qq) plots comparing the distribution of observed and expected p-values of tests carried out by OncodriveFML on mutations in 5' UTRs in 8 cohorts of tumors (from dataset WG-608) and the corresponding pan-cancer cohort. False discovery rate thresholds (0.1 and 0.25) are indicated as short red and green segments in each graph.

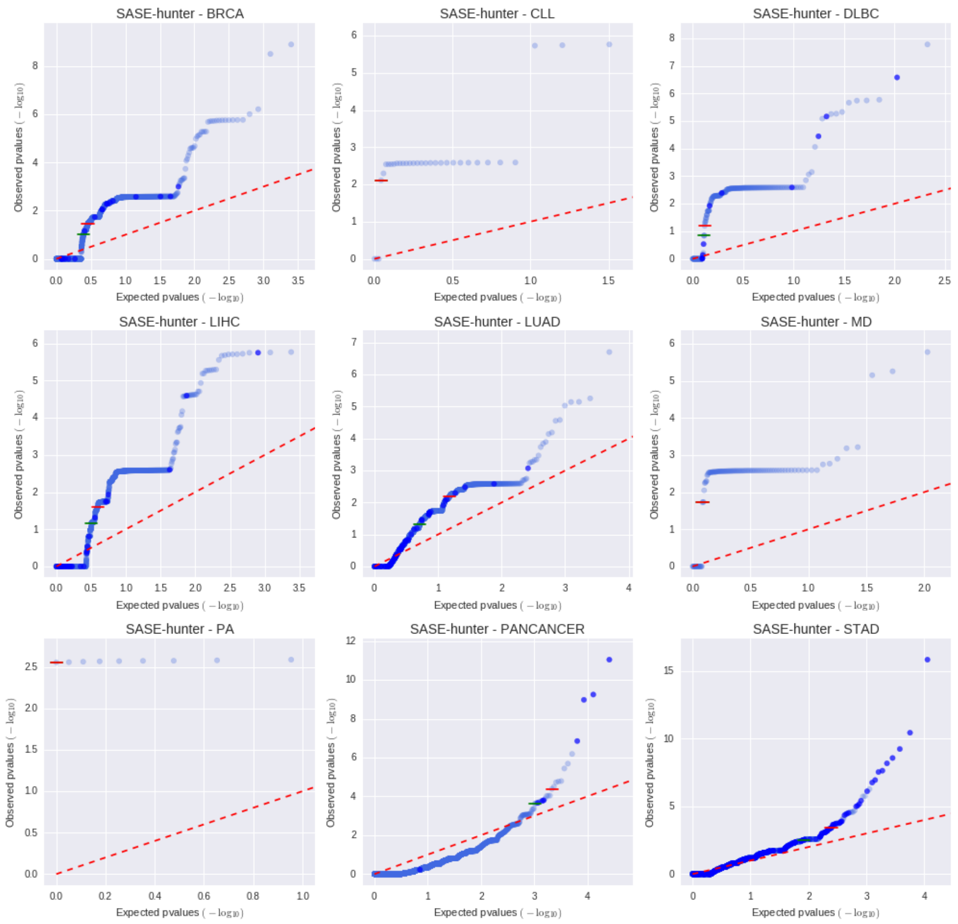

Figure 27. Quantile-quantile (qq) plots comparing the distribution of observed and expected p-values of tests carried out by SASE-hunter on mutations in splice intronic regions in 8 cohorts of tumors (from dataset WG-608) and the corresponding pan-cancer cohort. False discovery rate thresholds (0.1 and 0.25) are indicated as short red and green segments in each graph.

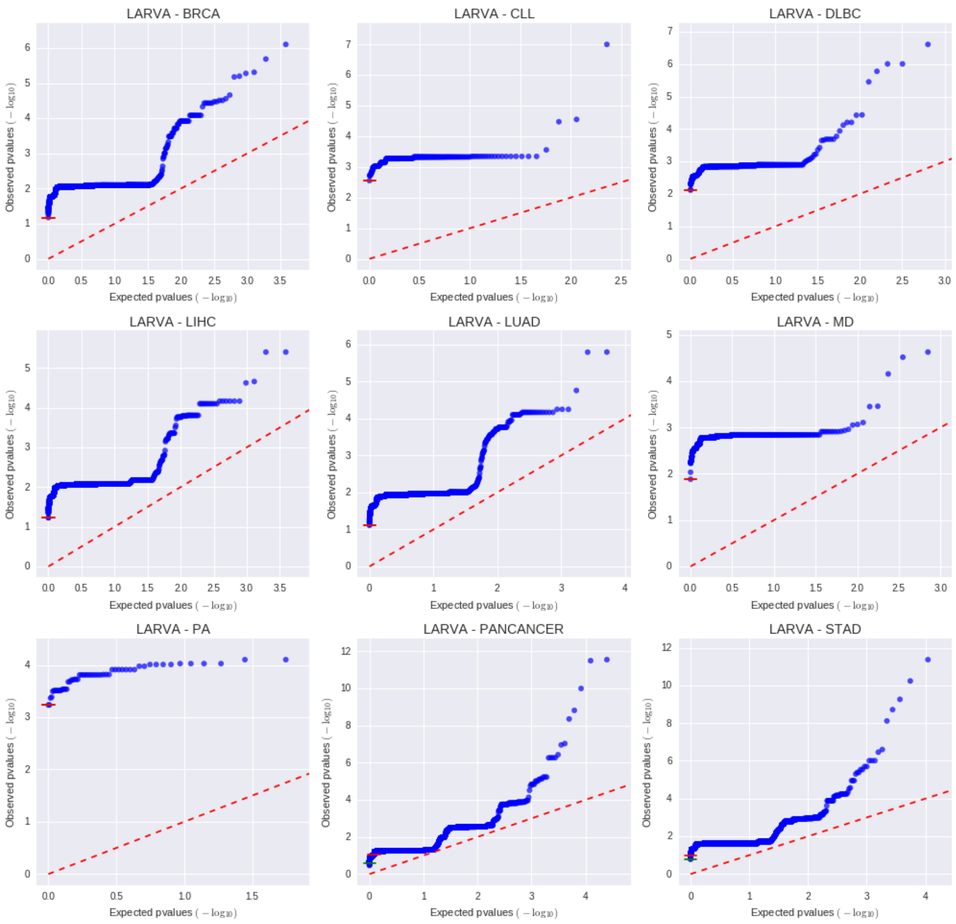

Figure 28. Quantile-quantile (qq) plots comparing the distribution of observed and expected p-values of tests carried out by LARVA on mutations in splice intronic regions regions in 8 cohorts of tumors (from dataset WG-608) and the corresponding pan-cancer cohort. False discovery rate thresholds (0.1 and 0.25) are indicated as short red and green segments in each graph.

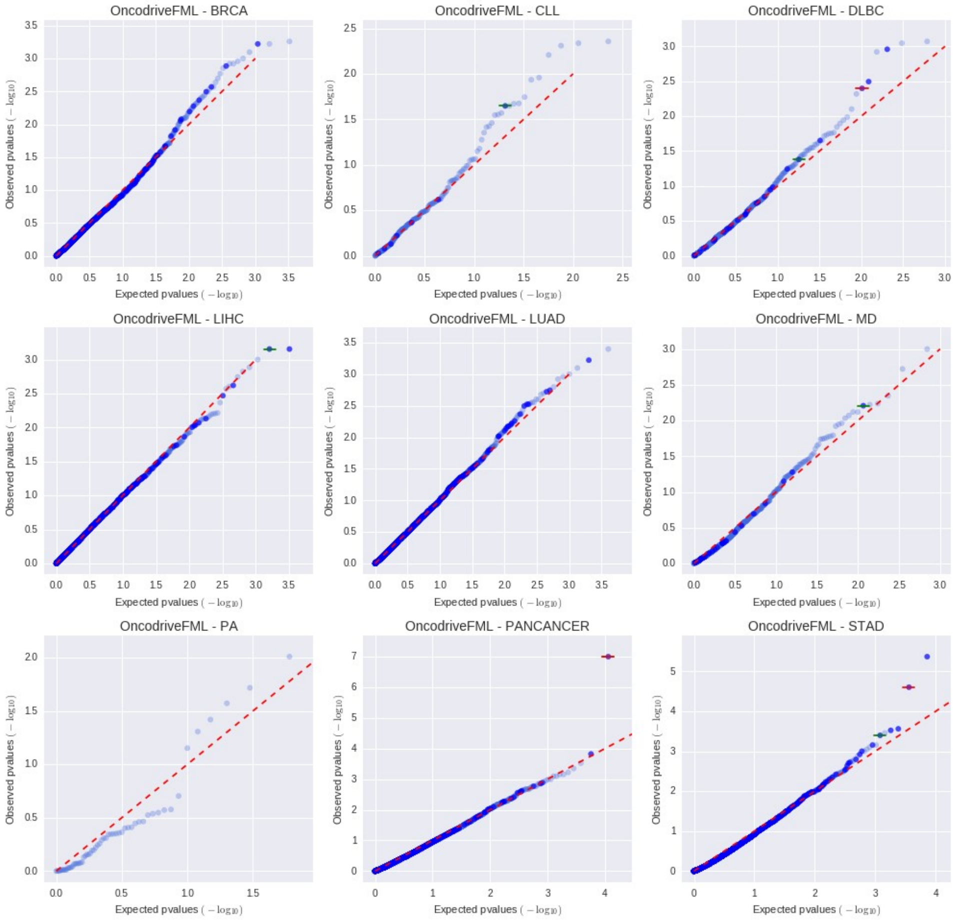

Figure 29. Quantile-quantile (qq) plots comparing the distribution of observed and expected p-values of tests carried out by OncodriveFML on mutations in splice intronic regions regions in 8 cohorts of tumors (from dataset WG-608) and the corresponding pan-cancer cohort. False discovery rate thresholds (0.1 and 0.25) are indicated as short red and green segments in each graph.

References

Gonzalez-Perez, A., & Lopez-Bigas, N. (2012). Functional impact bias reveals cancer drivers. *Nucleic Acids Research*, *40*(21). http://doi.org/10.1093/nar/gks743

Lawrence, M. S., Stojanov, P., Polak, P., Kryukov, G. V, Cibulskis, K., Sivachenko, A., … Getz, G. (2013). Mutational heterogeneity in cancer and the search for new cancer-associated genes. *Nature*, *499*(7457), 214–8. http://doi.org/10.1038/nature12213

Lochovsky, L., Zhang, J., Fu, Y., Khurana, E., & Gerstein, M. (2015). LARVA: An integrative framework for large-scale analysis of recurrent variants in noncoding annotations. *Nucleic Acids Research*, *43*(17), 8123–8134. http://doi.org/10.1093/nar/gkv803

Porta-Pardo, E., & Godzik, A. (2014). E-Driver: A novel method to identify protein regions driving cancer. *Bioinformatics*, *30*(21), 3109–3114. http://doi.org/10.1093/bioinformatics/btu499

Smith, K. S., Yadav, V. K., Pedersen, B. S., Shaknovich, R., Geraci, M. W., Pollard, K. S., & De, S. (2015). Signatures of accelerated somatic evolution in gene promoters in multiple cancer types. *Nucleic Acids Research*, *43*(11), 5307–5317. http://doi.org/10.1093/nar/gkv419

Tamborero, D., Gonzalez-Perez, A., & Lopez-Bigas, N. (2013). OncodriveCLUST: Exploiting the positional clustering of somatic mutations to identify cancer genes. *Bioinformatics*, *29*(18), 2238–2244. http://doi.org/10.1093/bioinformatics/btt395
